# Hif-1α Knockdown Reduces Glycolytic Metabolism and Induces Cell Death of Human Synovial Fibroblasts Under Normoxic Conditions

**DOI:** 10.1038/s41598-017-03921-4

**Published:** 2017-06-16

**Authors:** Manuel J. Del Rey, Álvaro Valín, Alicia Usategui, Carmen M. García-Herrero, María Sánchez-Aragó, José M. Cuezva, María Galindo, Beatriz Bravo, Juan D. Cañete, Francisco J. Blanco, Gabriel Criado, José L. Pablos

**Affiliations:** 1 Servicio de Reumatología, Instituto de Investigación Sanitaria Hospital 12 de Octubre (imas12), Madrid, Spain; 2 Universidad Complutense de Madrid, Madrid, Spain; 3 Departamento de Biología Molecular, Centro de Biología Molecular Severo Ochoa, Centro de Investigación Biomédica en Red de Enfermedades Raras (CIBERER), Instituto de Investigación Sanitaria Hospital 12 de Octubre (imas12), Universidad Autónoma de Madrid, Madrid, Spain; 4 Servicio de Cirugía Ortopédica y Traumatología, Hospital 12 de Octubre, Madrid, Spain; 5 Unitat d’Artritis, Servei de Reumatologia, Hospital Clínic de Barcelona and Institut d’Investigacions Biomèdiques August Pí i Sunyer, Barcelona, Spain; 6 Laboratorio de Investigación Osteoarticular y del Envejecimiento, Instituto de Investigación Biomédica de A Coruña, INIBIC, A Coruña, Spain

## Abstract

Increased glycolysis and HIF-1α activity are characteristics of cells under hypoxic or inflammatory conditions. Besides, in normal O_2_ environments, elevated rates of glycolysis support critical cellular mechanisms such as cell survival. The purpose of this study was to analyze the contribution of HIF-1α to the energy metabolism and survival of human synovial fibroblasts (SF) under normoxic conditions. HIF-1α was silenced using lentiviral vectors or small-interfering RNA (siRNA) duplexes. Expression analysis by qRT-PCR and western blot of known HIF-1α target genes in hypoxia demonstrated the presence of functional HIF-1α in normoxic SF and confirmed the glycolytic enzyme glyceraldehyde-3-phosphate dehydrogenase (GAPDH) as a HIF-1α target even in normoxia. HIF-1α silencing induced apoptotic cell death in cultured SF and, similarly, treatment with glycolytic, but not with OXPHOS inhibitors, induced SF death. Finally, *in vivo* HIF-1α targeting by siRNA showed a significant reduction in the viability of human SF engrafted into a murine air pouch. Our results demonstrate that SF are highly dependent on glycolytic metabolism and that HIF-1α plays a regulatory role in glycolysis even under aerobic conditions. Local targeting of HIF-1α provides a feasible strategy to reduce SF hyperplasia in chronic arthritic diseases.

## Introduction

It is generally assumed that high efficiency oxidative phosphorylation is the default source of ATP for most mammalian cells under normoxic conditions, whereas glycolysis is an emergency back-up to be used when oxygen levels are deficient^1^. However, the observation that lactate is regularly produced even in the presence of oxygen suggests that glycolysis is an active metabolic pathway under normal O_2_ conditions^2, 3^.

Aerobic glycolysis is considered a hallmark of the metabolic switch experienced by most cancer and immune cells undergoing activation that promotes the expression of pro-inflammatory factors and reduces apoptosis^4–9^. In contrast to the slower ATP production of the oxidative phosphorylation (OXPHOS) triggered by mitochondrial biogenesis, glycolysis can rapidly be activated via the induction of enzymes that are involved in this pathway, swiftly generating not only ATP, but also biosynthetic intermediates to support rapid cell growth and their specific effector functions.

Recent investigations have provided insight into the molecular mechanisms that trigger the shift to glycolysis during immune cell activation, showing a link between glycolysis and HIF-1α as a critical axe for the acquisition of an inflammatory phenotype. In macrophages stimulated with LPS, accumulation of succinate, an intermediate metabolite of the tricarboxilic acid cycle (TCA), suppresses the activity of the prolyl hydroxilase (PHD) and stabilizes hypoxia-inducible factor 1α (HIF-1α) protein, a transcription factor that is crucial for the induction of enzymes involved in glycolysis^10^. Furthermore, the pyruvate kinase PKM2, an enzyme that promotes metabolism of pyruvate to lactate, increases the transcriptional activity of HIF-1α and the transcription of key glycolytic enzymes and IL-1β^11^. This metabolic reprogramming mediated by HIF-1α orchestrates the inflammatory differentiation of immune cells. Thus for instance, deficiency in HIF-1α in T cells reduced the expression of the glycolytic molecules and altered the balance between Th17 and Treg cell lineages^12^. These studies further emphasize the link between HIF-1α and glycolysis for the induction of an inflammatory phenotype.

Similar to immune cells, fibroblastic cells such as rheumatoid arthritis synovial fibroblasts (RASF), human skin keloid fibroblasts or stromal cancer associated fibroblasts (CAF), have elevated glycolysis/OXPHOS ratios^2, 13, 14^, suggesting that glycolysis may contribute to support their activity and the progression of chronic inflammation. Interestingly, quiescent fibroblasts in normal O_2_ conditions, also exhibit high metabolic activity with elevated rates of glycolysis, pentose phosphate pathway, TCA and NADPH generation that support critical cellular mechanisms such as cell survival. Inhibition of the pentose phosphate pathway, which overflow fluxes back to glycolysis, results in apoptosis of primary human fibroblasts^3^, demonstrating an essential role for these metabolic pathways in normoxia. Recent studies have shown that, in normal O_2_ environments, fast activation of the glycolytic pathway may respond to rapid fluctuations in energy demands needed to maintain the adequate levels of ATP for cell survival and support rapid and diverse membrane changes required for cell movement^15, 16^. Manipulation of the energetic needs of the membrane transporters in different normal and cancer cell lines growing in normal O_2_ conditions led to changes in glycolytic metabolism with no significant changes in the oxygen consumption rate (OCR)^17^. Despite the essential role that glycolysis seems to play in fibroblast function, the molecular mechanisms regulating this metabolic pathway under normal O_2_ conditions still remain to be fully elucidated.

The aim of our study was to investigate the contribution of HIF-1α to the metabolic activity and survival of human SF under homeostatic conditions in normoxia. Our data demonstrate that HIF-1α regulates the expression of the glycolytic enzyme glyceraldehyde-3-phosphate dehydrogenase (GAPDH) and lactate production in fibroblasts cultured under normal O_2_ conditions, independently of changes in OXPHOS metabolism. Furthermore, inhibition of either HIF-1α or glycolysis strongly reduces fibroblasts survival rates. Our data support a critical role for HIF-1α in regulating glycolysis and SF survival under normoxic conditions, and therefore HIF-1α represents a potential target to reduce the fibroblastic hyperplasia that poses a relevant pathogenetic element of chronic arthritis.

## Results

### HIF-1α regulates the expression of the glycolytic enzyme GAPDH in normoxic SF

In order to investigate the potential role of HIF-1α in regulating the energy metabolism of fibroblasts in normoxia, we used a lentiviral vector system to stably express HIF-1α small-interfering RNA (siRNA) in SF.

**Figure 1:**
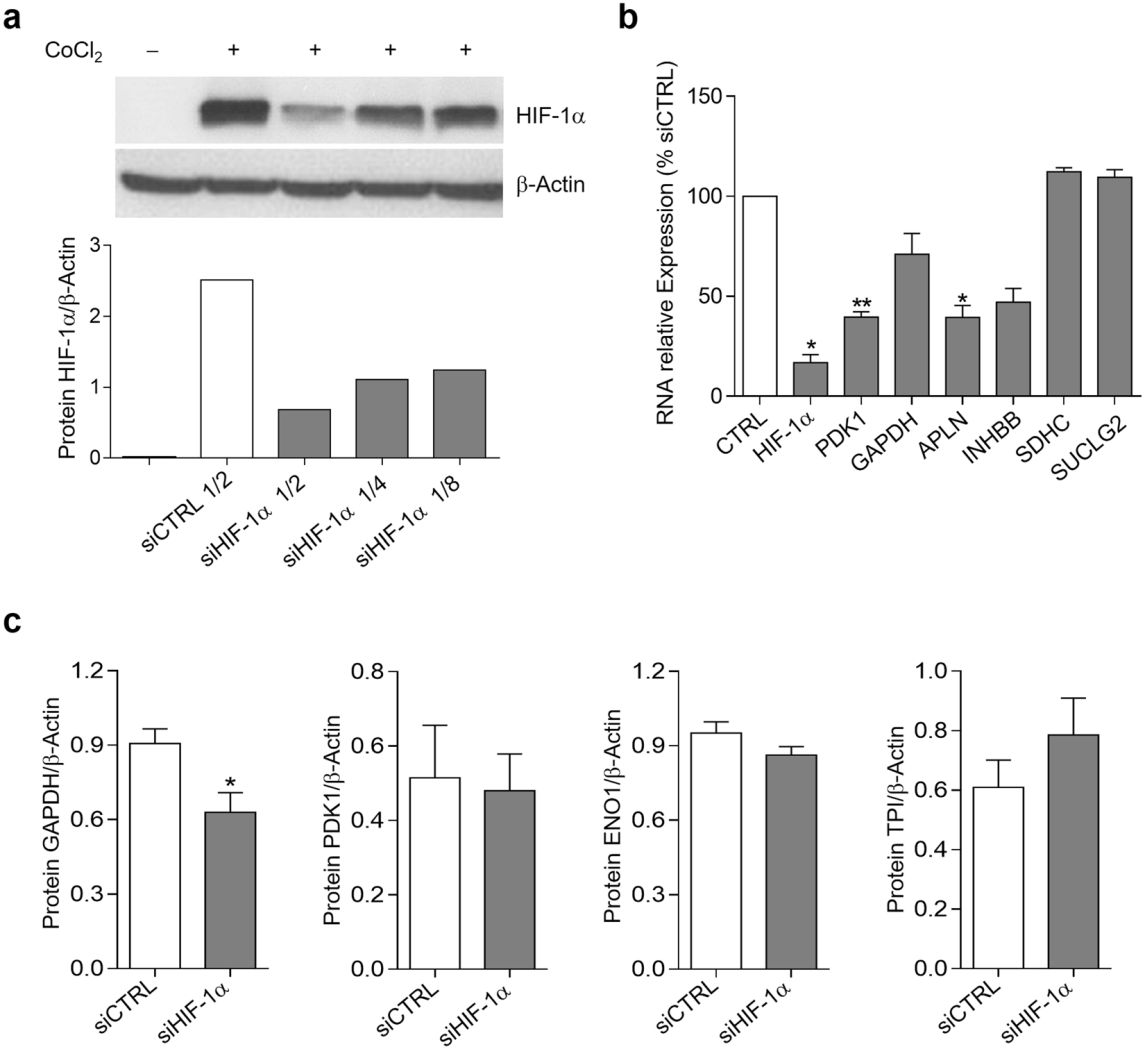
Evaluation of HIF-1α function in SF under normoxic conditions. (**a**) Titration of HIF-1α silencing in SF at 7 days after HIF-1α or control (CTRL) siRNA lentiviral transduction after treatment with 300 μM CoCl_2_. Western blot image of HIF-1α and β-actin, and densitometric analysis of HIF-1α expression normalized to the β-actin level. (**b**) Silencing efficiency of HIF-1α siRNA lentivirus in normoxia by qRT-PCR (siCTRL mRNA gene/β-actin ratio set to 100%). HIF-1α, PDK1, GAPDH, INHBB, SDHC and SUCLG2 genes were analyzed. Data are mean ± SEM of 6 independent experiments (*p = 0.03, **p = 0.015, Wilcoxon Signed Rank test). (**c**) HIF-1α knockdown down-regulates the expression of GAPDH. Protein expression of glycolytic enzymes GAPDH, PDK1, ENO1 and TPI under normoxic conditions measured by western blot in SF after lentiviral transduction with both HIF-1α and non-silencing control (CTRL) siRNA. Protein extracts were obtained 4 days after transduction. Data are mean ± SEM of 7 independent experiments (*p = 0.015, Wilcoxon test).

To check HIF-1α silencing efficiency we achieved the maximal expression of HIF-1α using CoCl_2_ in SF cultures to prevent HIF-1α degradation^18^. Upon knockdown of SF growing in normoxic conditions with specific HIF-1α siRNA we observed a strong down-regulation of HIF-1α protein compared to control siRNA fibroblasts (72.9% with the highest lentiviral titer, Fig. 1a). Previous investigations have demonstrated very low levels of HIF-1α protein under normal O_2_ conditions in transformed cell lines^19, 20^. For the purpose of evaluating the potential role of HIF-1α activity in normoxia, we analyzed the mRNA expression of target genes known to be regulated by HIF-1α in hypoxia^21, 22^. In addition to the strong down-regulation of HIF-1α mRNA (83.2 ± 4%, mean ± SEM), we found a considerable down-regulation of HIF-1α-regulated genes such as pyruvate dehydrogenase kinase 1 (PDK1) (60.4 ± 2.8%, mean ± SEM), GAPDH (29 ± 10.4%, mean ± SEM), apelin (APLN) (60.6 ± 6%, mean ± SEM) and inhibin beta (INHBB) (52.9 ± 6.9%, mean ± SEM). In contrast, the expression of genes not regulated by HIF-1α, such as succinate dehydrogenase (SDHC) or succinyl-CoA ligase (SUCLG2) was not altered (Fig. 1b), thus confirming the specificity of HIF-1α knockdown. Protein levels of GAPDH were also significantly down-regulated upon HIF-1α silencing in SF, but not those of PDK1, enolase 1 (ENO1) or triosephosphate isomerase (TPI) (Fig. 1c). Conversely, stabilization of HIF-1α protein by CoCl_2_ induced the expression of GAPDH both at mRNA and protein level in parallel to the HIF-1α protein stabilization (Supplementary Information file (Fig. S1)).

These data support the presence of functional HIF-1α protein in normoxia that regulates the expression of the glycolytic enzyme GAPDH but has a lesser impact on other HIF-1α target genes.

To explore additional metabolic or cell survival pathways potentially regulated by HIF-1α in normoxia, we used a proteomic approach by iTRAQ labelling. By applying a cut-off of unused ProtScore >1.3, we identified 321 proteins in the SF cell extracts. We selected proteins with two criteria: 1) fold change siHIF-1α (HIF) versus non-silencing (CTRL) siRNA treated SF <0.6 for down-regulated or >2 for up-regulated proteins in at least one of duplicate experiments, and 2) both comparisons show a similar sense of regulation (up- or down-). Down-regulated proteins pertained to energy metabolism and glycolysis, but changes of variable sign in proteins potentially involved in apoptotic or cell proliferation processes were also identified (Supplementary Information file (Tables S1 and S2)). Although this small size analysis precludes reaching statistical significant conclusions, data are consistent with a role for HIF-1α on the regulation of the glycolytic pathway in normoxic SF.

### The balance glycolysis/OXPHOS is shifted after HIF-1α silencing in SF

**Figure 2:**
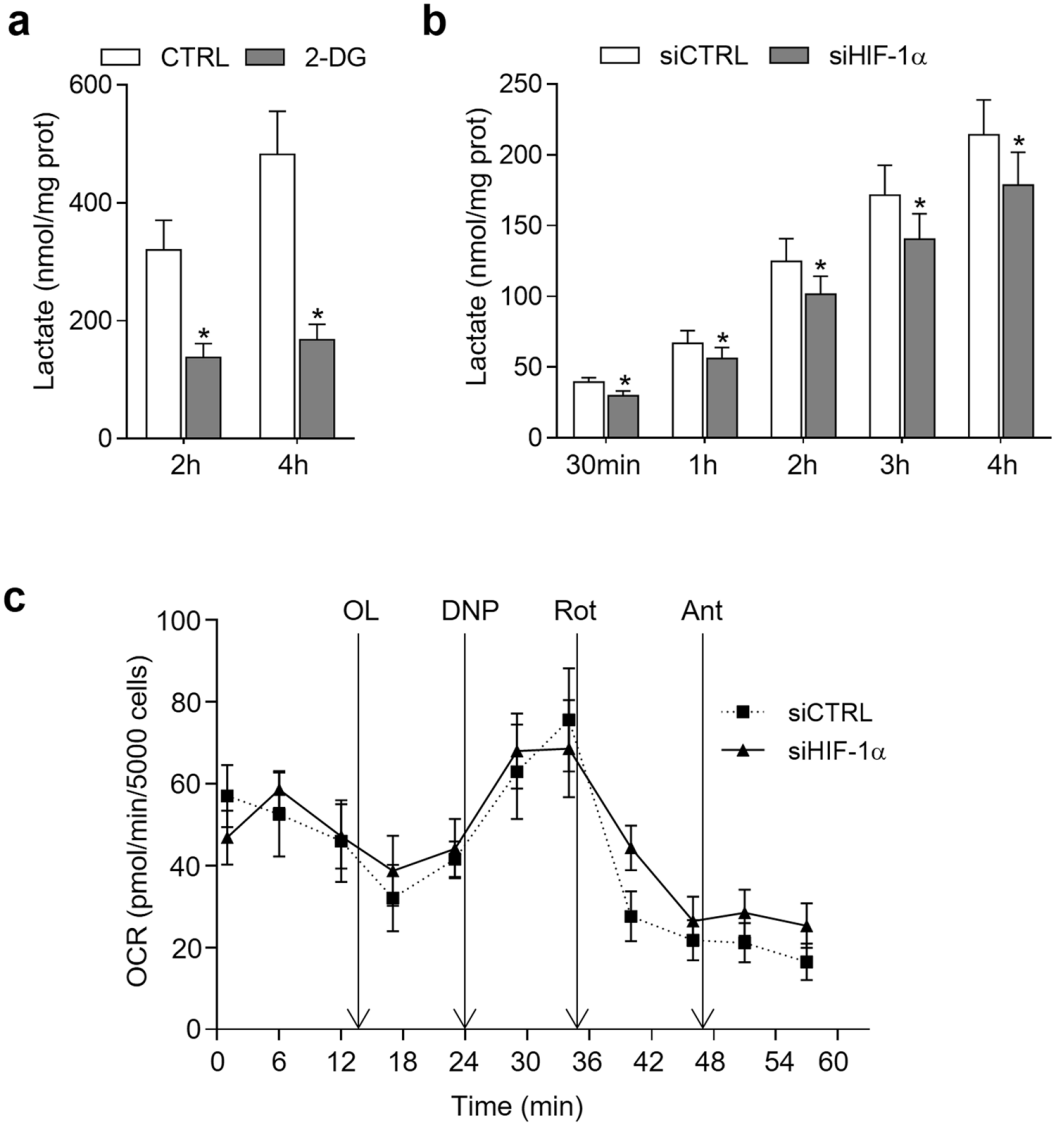
Analysis of energy metabolism in SF. (**a**) Lactate production by SF (n = 6) at 2 h and 4 h after 2-DG treatment (40 mM) (*p = 0.03 in all comparisons, Wilcoxon test) (Mean ± SEM are indicated). (**b**) Lactate production in HIF-1α silenced and non-silenced (CTRL) SF cultures at 30 minutes, 1 h, 2 h, 3 h and 4 h after media replenishing. Data represent mean ± SEM of 3 independent experiments (*p = 0.03 in all comparisons, Wilcoxon test). (**c**) Real-time analysis of OCR in HIF-1α or non-silencing (CTRL) siRNA lentiviral transduced SF after the sequential addition of OL, 2,4-dinitrophenol (DNP), rotenone (Rot) and antimycin (Ant) to the cells. Results are representative of 5 replicates from 2 independent experiments (Mean ± SEM).

To further investigate the contribution of HIF-1α to the regulation of the aerobic glycolysis in SF, we determined the amount of lactate released to the medium as the final product of glycolytic flux. The glycolytic inhibitor 2-deoxy-D-glucose (2-DG)^23^ strongly reduced the levels of lactate released by SF in normoxia (Fig. 2a), indicating that aerobic glycolysis is an active metabolic pathway in these cells. In agreement with the down-regulated expression of the glycolytic enzyme GAPDH described above, HIF-1α silencing in SF significantly decreased the lactate production (Fig. 2b), suggesting that HIF-1α regulates the glycolytic pathway in normoxic SF. In contrast, we did not detect significant changes in the aerobic mitochondrial respiration activity, determined as OCR, in HIF-1α-silenced SF (Fig. 2c).

Basal OCR in SF was relatively low (below 70 pmol/min/5000 cells) (Fig. 2c) compared to other cell types such as LC5 cells (data not shown), or to that reported in T cells, macrophages or cancer cells^24–26^. As expected, the addition of oligomycin (OL), an OXPHOS inhibitor, decreased OCR levels below the basal line, suggesting an active oxidative phosphorylation concomitant to the observed glycolytic activity. We did not find differences between HIF-1α and control siRNA SF in the oligomycin sensitive respiratory rate (OSR) (11.5 ± 7.1 and 16.5 ± 3.5 pmol O_2_/min/5000 cells respectively, mean ± SEM). Mitochondrial uncoupling with DNP increased the oxygen consumption in both siRNA-transduced SF, and inhibition of the mitochondrial electron transport chain with rotenone and antimycin, induced a strong decrease of OCR. These responses were similar in HIF-1α knockdown and control SF (Fig. 2c) indicating that, in contrast to glycolysis, HIF-1α does not regulate mitochondrial respiration in SF. Moreover, the reduction of the glycolytic activity detected upon HIF-1α silencing is not coupled to an increase in OXPHOS, suggesting that both metabolic pathways are independently regulated.

These results point to HIF-1α as an important regulator of SF glycolysis in normoxia, modulating the balance between glycolysis and mitochondrial activity in these cells.

### HIF-1α knockdown and glycolysis inhibition induce apoptotic cell death in fibroblasts

**Figure 3:**
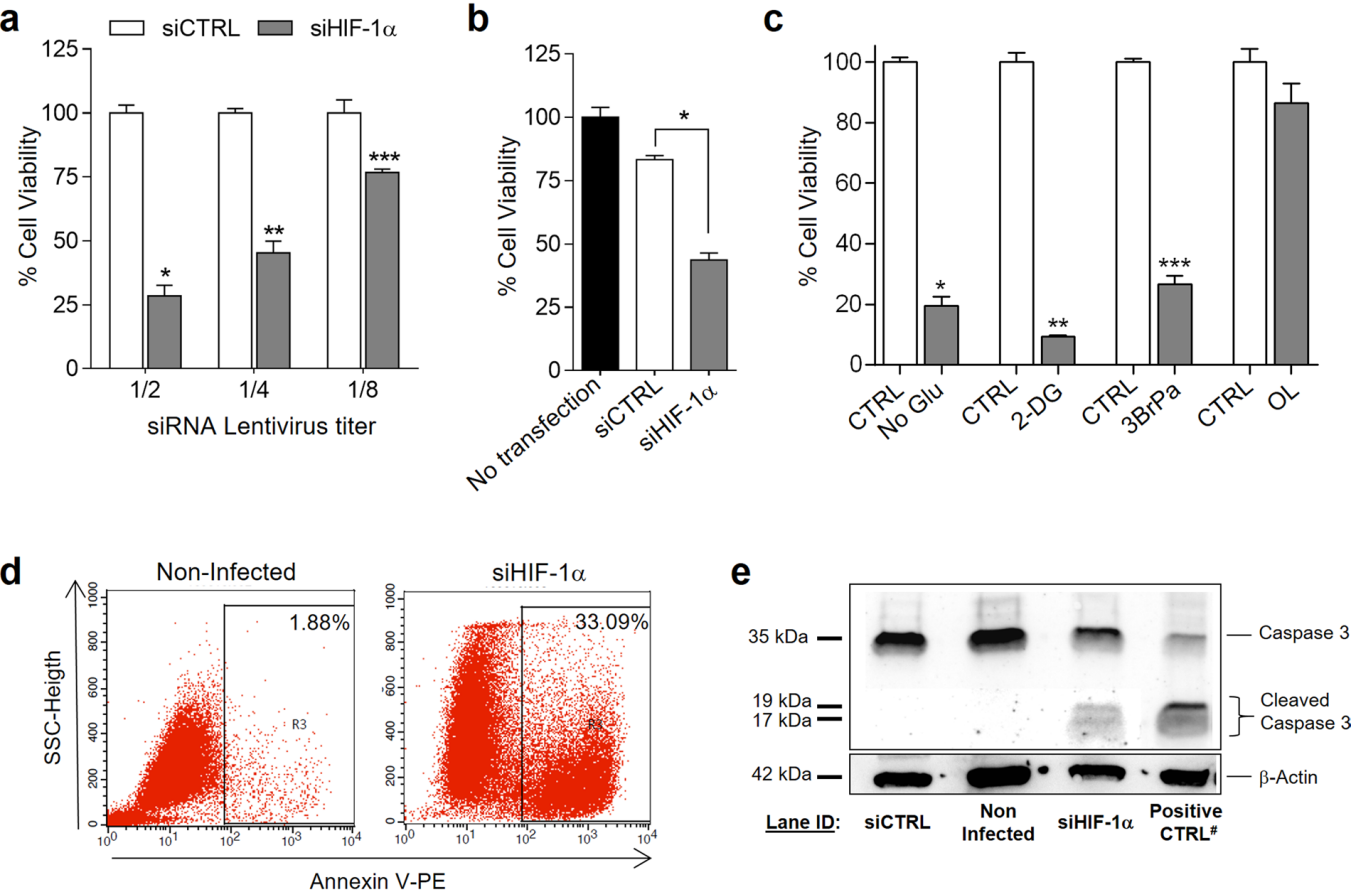
HIF-1α knockdown and glycolysis inhibition induce apoptotic cell death in fibroblasts. (**a**) SF cell viability after HIF-1α knockdown 7 days after transduction (siCTRL transduced SF considered 100%). Results are representative of 6 SF lines and mean ± SEM are represented (*p < 0.0001, **p = 0.0004, ***p = 0.002, Mann-Whitney U-test) (**b**) SF viability after transfection with HIF-1α siRNA duplexes at 72 h post-transfection (representative of 2 SF lines; Mean ± SEM are represented) (*p = 0.016, Mann-Whitney U-test). (**c**) Cell viability in SF after cultured in non-glucose medium (No Glu) (n = 8), and after treatment for 8 h with 2-DG (40 mM) (n = 7), 3BrPa (25 μM) (n = 4) and OL (6 μM) (n = 4). Data represent mean ± SEM (*p < 0.0001, **p = 0.0001, ***p = 0.001, Wilcoxon test). (**d**) Flow cytometry analysis of apoptotic cell death following Annexin V-PE staining. Representative histogram of HIF-1α silenced SF is shown. Lentiviral titer used is 1/2. (**e**) Representative western blot of caspase-3 and cleaved caspase-3 in DF using β-actin as an endogenous control. ^#^Positive CTRL: LC5 cells treated with staurosporine 1 μm for 3 h. Full-length blots are presented in Supplementary Information file (Fig. S3).

Fibroblasts transduced with HIF-1α and control scrambled siRNA lentiviral particles and maintained in culture for more than 5 days in normoxic conditions showed a dramatic reduction in cell viability. We observed that the reduction in cell viability correlated with lentiviral titers (Fig. 3a). Similar results were observed in cultures of adult human fibroblasts derived from different tissue locations such as lung (human pulmonary fibroblasts-HPF) or skin (dermal fibroblasts-DF). In contrast, viability of the epithelial cell line HEK 293T was not affected (data not shown). Cell viability was similarly reduced in hypoxia (0.5% O_2_) under maximal HIF-1α expression conditions (data not shown). These data support an important role for HIF-1α as regulator of human adult fibroblast survival, regardless their status or tissue origin.

To confirm that the effect was dependent on siRNA knockdown and not the lentiviral construct, we silenced HIF-1α in SF lines by transfection of siRNA duplexes with different HIF-1α siRNA sequences. Similar to the results obtained with the lentiviral-mediated HIF-1α knockdown, we observed a significant decrease in cell viability 72 h after transfection of SFs with HIF-1α siRNA compared to control siRNA (Fig. 3b).

To mimic the effect of HIF-1α knockdown on the regulation of GAPDH expression and aerobic glycolysis, SF were cultured in non-glucose medium, or treated with either 2-DG or the GAPDH inhibitor 3-bromopyruvate (3BrPa)^27^. Interestingly, inhibition of glycolysis with any of the above treatments induced cell death in SF (Fig. 3c). In contrast, OXPHOS inhibition with oligomycin did not have any significant effect on cell viability (Fig. 3c). These data suggest that, in SF, aerobic glycolysis is critical for cell survival.

To investigate the potential mechanism of cell death, apoptosis analyses were performed in SF. By flow cytometry, we observed a high percentage of Annexin V positive cells in HIF-1α siRNA transduced fibroblasts compared to control siRNA fibroblasts from different sources (Fig. 3d). Apoptotic cell death induced by HIF-1α silencing was confirmed by detection of the effector caspase-3 cleavage (Fig. 3e). These data confirm that HIF-1α knockdown induces apoptosis in normoxic SF cultures.

Collectively, these data point to a high dependence of SF on glycolytic metabolism. For this reason, glycolysis down-regulation by HIF-1α silencing may lead to apoptotic cell death in SF.

### HIF-1α silencing decreases SF viability *in vivo*

To evaluate the SF sensitivity to the siRNA-mediated knockdown of HIF-1α *in vivo*, we used a model of human SF graft into an air pouch membrane in NOD *scid gamma* mice (NSG) as a model of inflammation that simulates the structure of the synovial membrane and cavity^28, 29^. Since all SF exhibited a similar response to HIF-1α silencing, we used SF from rheumatoid arthritis (RA) patients.

**Figure 4:**
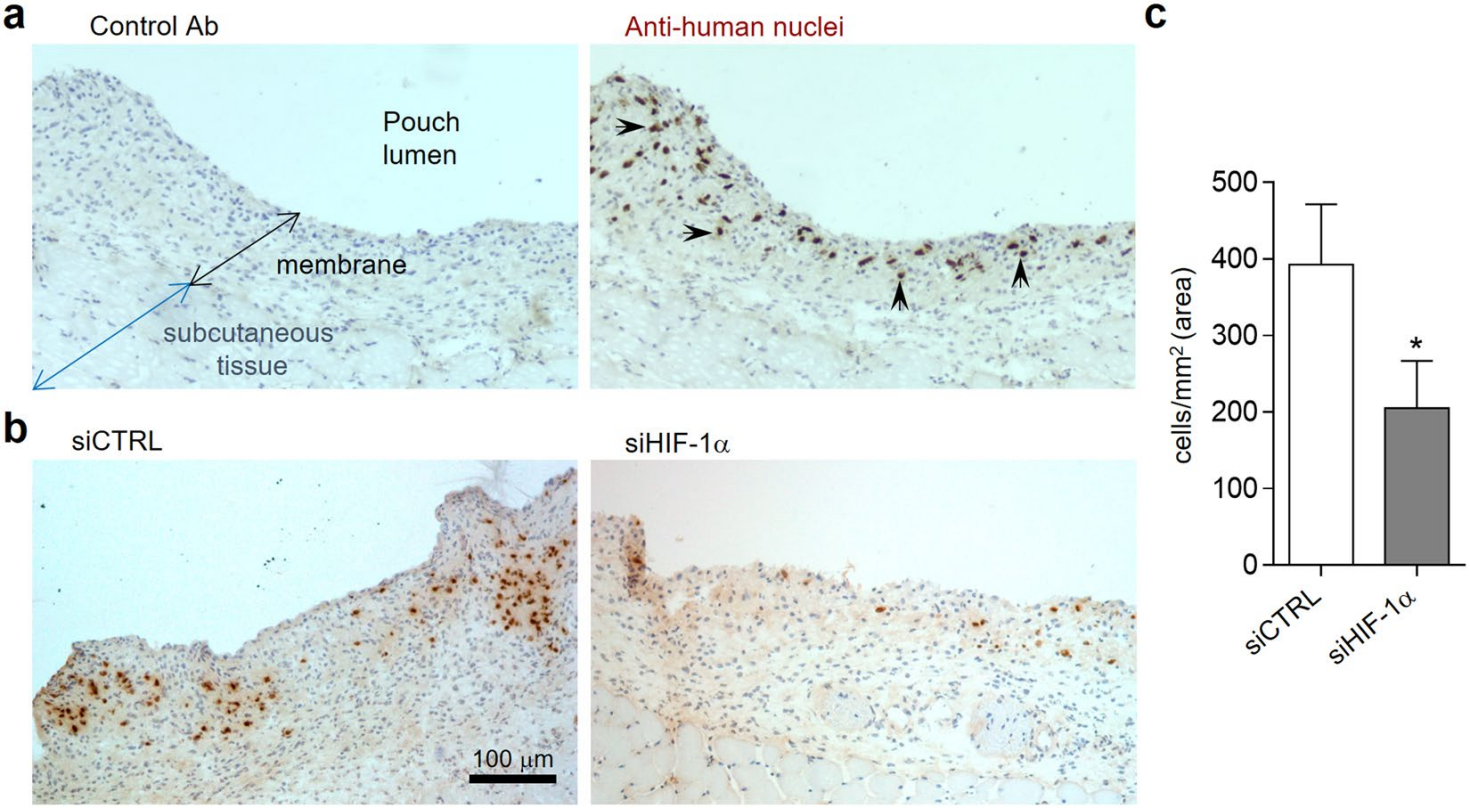
Viability of implanted RASF in the air pouch after HIF-1α siRNA injection. (**a**) IHC immunoperoxidase labelling with isotype control (left) or anti-human nuclei (right), identifying RASF in the air pouch wall at day 7 after implant. Examples of positive human nuclei marked by arrows. (**b**) Representative human nuclei staining in sections from siCTRL and siHIF-1α injected air pouches (**c**) Quantitative analysis of human cell nuclei per area (mm^2^) in siCTRL and siHIF-1α air pouch membranes. Mean ± SEM data are of 9 mice per group (*p = 0.04, Mann-Whitney U-test).

RASF injected in the air pouch remained engrafted 7 days after injection within the synovial membrane-like structure that lined the air pouch cavity, as demonstrated by specific immunolabelling of human nuclei (Fig. 4a). HIF-1α and control siRNA duplexes were injected into the air pouches, and the density of human RASF was determined two days later. The density of human nuclei was significantly reduced in the membranes of HIF-1α siRNA injected mice compared to control treated mice (205 ± 62 and 393 ± 79 cells/mm^2^ respectively, mean ± SEM) (Fig. 4b,c). These results suggest that treatment with HIF-1α siRNA also induces cell death of SF *in vivo*.

## Discussion

Cells modulate their metabolism to adapt to different energy requirements and signaling events in physiological situations. It has long been assumed that in conditions of normal tissue oxygen pressure the main energy source of the cells is the oxidative phosphorylation, while glycolysis is less efficient. Recent findings showed that aerobic glycolysis can be a metabolic alternative of normal cells and that its role is not limited to supporting proliferation^3, 15–17^. While progress has been made in understanding the mechanisms regulating the metabolic shift to glycolysis under hypoxia, little is known about the mechanisms that control this metabolic pathway in normal O_2_ environment, as well as the cellular processes demanding energy from aerobic glycolysis.

HIF-1α is a master regulator of metabolism known to control glycolysis in response to hypoxic stress, through the transcriptional regulation of genes encoding glucose transporters and glycolytic enzymes^30, 31^. However, increased glycolysis and HIF-1α activity may also occur under aerobic conditions as demonstrated in cancer cells^4, 32^, and in the inflammatory differentiation of immune cells such as lymphocytes, myeloid cells and different type of fibroblasts^2, 12–14, 25^.

The glycolytic enzyme GAPDH contains a hypoxia responsive element (HRE) in its promoter which has been confirmed as a HIF-1α transcriptional target under hypoxic conditions^33–36^. Our work provides evidence that supports a role for HIF-1α as a regulator of glycolysis through GAPDH expression in SF under low HIF-1α expression conditions in normoxia. HIF-1α silencing in fibroblast led to down-regulation of genes implicated in the metabolism of glycolysis and ultimately the inhibition of the glycolytic activity in these cells. Furthermore, we have shown that HIF-1α and glycolysis are essential for fibroblasts survival even in normal O_2_ conditions.

Although the mechanism of oxygen-dependent post-translational regulation of HIF-1α is well understood, a number of reports indicate that HIF-1α protein is regulated and partially stabilized also in normoxia, suggesting that this transcription factor may also be critical for cellular functions others than in hypoxia^37–40^. After HIF-1α knockdown with specific siRNAs in SF, we observed a significant decrease in the expression of known HIF-1α-target genes under normoxic conditions. Expression of HIF-1α protein in normoxia has been reported in transformed cell lines^19, 20^. However, we and others could not detect HIF-1α protein in primary human fibroblasts^41, 42^, a finding that reflects the technical limitations of using normal primary cells. Although we failed to detect HIF-1α protein in our cellular system, the silencing of HIF-1α with siRNAs against different sequences and delivered through different approaches yielded similar outcomes, thus ruling out potential off target effects. Together with the specificity of the genes modulated upon silencing of this transcription factor, our data support the presence of functional levels of HIF-1α protein in fibroblasts.

In contrast to the robust effect observed in several HIF-1α target genes at the transcriptional level, we only detected a significant inhibition of GAPDH protein levels upon HIF-1α silencing. These results may reflect differences in the sensitivity of both technical approaches, or point out to a high stability of these proteins, that make significant changes at the transcriptional level not enough for an impact in the translation to protein. However, we observed that both HIF-1α silencing and glycolytic inhibitors induced a significant reduction in lactate production, demonstrating that HIF-1α has a significant impact on the glycolytic metabolism of SF growing in normal O_2_ environment. Thus, our data stay in line with previous investigations and support the presence of low levels of functional HIF-1α that modulates glycolysis under normal O_2_ conditions. Further analysis of the enzymes involved in glycolysis and potentially regulated by HIF-1α will be necessary to identify the specific pathways regulated by this transcription factor in normoxia.

Recent investigations suggest a demand-driven model of energy use in which glycolysis and oxidative phosphorylation are not necessarily opposite metabolic pathways, but in fact complementary production modes, supplying ATP and other biosynthetic molecules to independent cellular processes with different timescales of energy demands. In this regard, Epstein *et al*. showed that inhibition or activation of transporters on the cell membrane of different normal and cancer cells led to reduction or increase in glycolytic activity respectively, while the respiration rate remained unchanged^17^. Interestingly, we did not detect significant changes on the OCR levels, supporting a specific role for HIF-1α regulating the glycolytic metabolism.

Fibroblasts are not a unique example of cells with high level of glucose uptake, even in a quiescent state. A specific role for glycolysis independent of OXPHOS has been also demonstrated in T cells where, even though OXPHOS and aerobic glycolysis interchangeably fuel T cell proliferation and survival, only aerobic glycolysis can facilitate their full effector function^9^. Moreover, low mitochondrial potential hematopoietic stem cells use glycolysis instead of mitochondrial respiration for their energy demands, associated with the normoxic up-regulation of HIF-1α and its downstream target genes^43^. Our results therefore support the view of independent regulatory pathways for OXPHOS and glycolysis and the role of HIF-1α as an important factor modulating specifically the glycolytic metabolism in normal O_2_ environment.

It has been shown that redirecting energy metabolism towards glycolysis, a process mostly mediated by HIF-1α, minimizes oxidative damage and suppresses apoptosis in hypoxic conditions^8, 44^. Interestingly, under normal oxygen conditions, we observed an increase in apoptotic markers and a significant reduction of cell viability in HIF-1α silenced SF, both in cell cultures and *in vivo*. A similar decrease in cell viability occurs in the presence of glycolytic inhibitors such as 2-DG or 3BrPa but not with olygomycin, an inhibitor of the oxidative metabolism. Recent studies have shown that in normoxia, failure of glycolysis to quickly respond to specific energy demands of the cell may cause lethal cell damage^17^. In this regard, our data suggest that after HIF-1α silencing, the decrease of quick energy from glycolysis may give rise to a defective response to provide energy to specific cellular processes, ultimately leading to cell death. This mechanism may not be exclusive, since iTRAQ analysis identified additional proteins, in line with previous data on HIF-1α regulation of multiple potentially involved in cell proliferation and survival processes^45^. Additional studies will be necessary to fully establish the link between aerobic glycolysis and apoptosis in normoxia, as well as additional biological processes potentially regulated by HIF-1α.

Since all tested fibroblasts, independently of their normal or pathologic condition or tissue origin, were sensitive to HIF-1α silencing-induced cell death, our findings provide an opportunity to target fibroblast hyperplasia, a common factor to different pathologic conditions. HIF-1α-targeting agents are being developed as potential anti-tumoral or anti-inflammatory therapies. However, systemic effects and toxicity of HIF-1α antagonists represent an important drawback^46^. We propose an alternative local approach in a setting of inflammatory fibroblast hyperplasia reminiscent of that observed in chronic arthritis. In the air pouch model, where human SF showed a distribution similar to human synovial lining, reduction of SF survival by HIF-1α siRNA injection demonstrates the feasibility of a local intra-articular administration. This approach reduces the toxicity associated to small molecule antagonists and the potential deleterious effects of systemic HIF-1α targeting. Furthermore, the observed dependency of SF survival on the aerobic glycolytic metabolism regulated by HIF-1α points to the inhibition of this metabolic pathway as an attractive alternative intervention to target inflammatory fibroblast hyperplasia.

All together our investigations provide insights into the mechanisms that control the glycolytic metabolism in normal O_2_ conditions, supporting a critical role for the transcription factor HIF-1α in homeostatic cellular biology of SF.

## Methods

### Cells and reagents

SF cultures were established by explant growth of small arthroscopic biopsy fragments of synovial tissues obtained from the knee of patients without previous joint disease at elective arthroscopy for minor traumatic lesions, or patients with RA or osteoarthritis (OA) at the time of prosthetic replacement surgery. Human adult pulmonary fibroblasts (HPF) were purchased from ScienCell Research Laboratories (Carlsbad, CA, USA). Normal skin dermal fibroblasts (DF) were established by explant growth of skin biopsies obtained from healthy individuals during minor skin surgery. All fibroblast cultures were maintained in 10% fetal calf serum (FCS)-DMEM medium (Lonza, Verviers, Belgium). Patients signed a written informed consent, and the study was approved by the Ethics Committee of Hospital 12 de Octubre, Madrid, Spain. All methods involving humans were performed in accordance with the relevant guidelines and regulations.

Where indicated, cells were treated for 8 h with inhibitors 40 mM 2-DG, 25 μM 3BrPa, or 6 μM OL (Sigma-Aldrich, Madrid, Spain).

### Oligo- and Lentivirus-Mediated siRNA Knockdown

Lentiviral transduction of fibroblasts with HIF-1α and control scrambled siRNA was carried out as previously described^47^. For the silencing of HIF-1α with siRNA duplex transfection, we used HIF-1α and control scrambled siRNA duplexes from Santa Cruz Biotechnology (Santa Cruz Biotechnology Inc, Santa Cruz, CA, USA) transfected with Lipofectamine 2000 (Invitrogen, Carlsbad, CA, USA).

### Quantitative Real Time-PCR (qRT-PCR) analyses

Total RNA was extracted using TRI Reagent (Invitrogen) according to the manufacturer’s protocol. For the quantification of mRNA, 1 μg was used for first strand cDNA synthesis with High Capacity cDNA Transcription Kit (Applied Biosystems, Foster City, CA, USA).

For PCR amplification we used cDNA and primers added to Power Sybr Green PCR Master Mix (Applied Biosystems). The sequences of PCR primers used are listed in Supplementary Information file (Table S3). β-actin was used as an endogenous reference. qRT-PCRs were performed on an Applied Biosystems 7500 Fast Real-Time PCR System (Applied Biosystems) or a Roche LightCycler 480 II (Roche Diagnostics, Mannheim, Germany) instrument. For relative quantification we compared the amount of target normalized to the endogenous reference using 2^−∆∆Ct^ formula (Ct = threshold cycle) or standard curves.

### Protein expression analysis

Total protein (30 μg) from control and HIF-1α-silenced SF growing for 4 days post-transduction in culture was extracted using lysis buffer (10 mM Tris-HCl pH8.0, 150 mM NaCl, 0.1% SDS, 1 mM EDTA pH8.0 and protease inhibitors) were separated by SDS-PAGE. Western blots were probed with following antibodies: mouse monoclonal anti-HIF-1α (BD Biosciences Pharmingen, San Jose, CA), mouse monoclonal anti-GAPDH-HRP (Abcam, Cambridge, UK), rabbit monoclonal anti-PDK1 (Abcam), rabbit monoclonal anti-TPI (Abcam), rabbit monoclonal anti-ENO1 (Abcam), rabbit polyclonal anti-caspase-3 (Cell Signaling Technology, Danvers, MA, USA) and anti-β-actin (Sigma-Aldrich). The protein bands were detected by enhanced chemiluminescence system (Bio-Rad Laboratories, Hercules, CA, USA) in an ImageQuant LAS4000 (GE Healthcare, Buckinghamshire, England) and quantified by densitometry using the ImageJ software. Values were normalized to the β-actin levels as endogenous reference.

Proteomic analysis was performed by iTRAQ labelling. Duplicate control and silenced HIF-1α of a single SF line were collected at 4 days post-infection. Total proteins were extracted using DIGE buffer (30 mM Tris, 8 nM urea, 2 mM thiourea and 4% CHAPS) by sonication. A 100 μg aliquot of protein from each sample was digested with trypsin and iTRAQ labelled according to the supplier’s instructions (ABSciex, Foster City, CA, USA). iTRAQ-labelled peptides were mixed and desalted prior to liquid chromatography coupled to mass spectrometry (LC-MS) analysis^48^.

### Analysis of lactate production and cellular respiration

Energy metabolism in control or HIF-1α-silenced SF was determined using the oxygen consumption rate (OCR) as an indicator of aerobic mitochondrial respiration, oligomycin sensitive respiratory rate (OSR) as indicator of OXPHOS and lactate concentrations in the culture medium as an index of aerobic glycolysis.

OCR was measured with a XF24 Extracellular Flux Analyzer (Seahorse Bioscience, Billerica, MA, USA). SF were seeded in XF 24-well cell culture microplates for 24 h. Assays were initiated by replacing cultures with fresh medium and cells were incubated for 2 h to allow media temperature and pH to reach equilibrium. After an OCR baseline measurement, OL, 2,4-dinitrophenol (DNP), rotenone and antimycin solutions were sequentially added, and changes in the OCR were analyzed as previously described^49^. OSR was calculated as the difference between basal OCR and OCR before adding OL.

Extracellular lactate was measured with the Lactate colorimetric assay kit II (Biovision, Milpitas, CA, USA). Briefly, SF were seeded in 6-wells plates (10^5^ cells/well). After overnight incubation, cells were washed with PBS and replenished with 1% FCS-medium fibroblasts, the supernatants were collected at the indicated time points (30 minutes, 1, 2, 3 and 4 h), and lactate levels were quantified by the colorimetric assay, according to the manufacturer’s instructions

### Analysis of cell viability in fibroblast cultures

Cell viability was analyzed in fibroblasts after transduction with HIF-1α siRNA or treatment with glycolytic inhibitors by Alamar Blue assay (Invitrogen, Camarillo, CA, USA) either 72 h after transfection with siRNA duplexes, 7 days after lentivirus transduction, or 8 h after treatment with inhibitors of the energy metabolism.

Apoptosis was evaluated in HIF-1α-silenced fibroblasts by flow cytometry using PE-Annexin V Apoptosis Detection Kit (Biolegend Inc., San Diego, CA, USA). Cells were analyzed on a BD FACSCalibur instrument (Becton Dickinson, San José, CA, USA). Cleaved caspase-3 was also detected by western blot as described above using anti-caspase-3 antibody.

### Humanized air pouch model

Animal experiments followed institutional guidelines and were approved by Animal Care and Use Committee of Hospital 12 de Octubre with protocol reference PROEX 407/15. Air pouches were generated on the back of 8- to 10-week-old NOD *scid gamma* (NOD.Cg-*Prkdc* ^*scid*^ *Il2rg* ^*tm1Wjl*^/SzJ) (NSG^TM^) mice by subcutaneous injection of 3 ml sterile air on day 0 and were maintained by reinjecting 2 ml of sterile air on day 3. On day 4, RASF suspensions (2 × 10^6^ cells in 0.5 ml PBS) were injected into the air pouch cavity. At day 9, 15 μg of HIF-1α or control scrambled siRNA duplexes combined with Invivofectamine reagent (Invitrogen) in 200 μl of 5% glucose were injected into the air pouch cavity. Mice (n = 9 per group) were sacrificed on day 11 and the air pouch cavity membrane together with the subcutaneous tissues was dissected and snap-frozen in OCT compound (Sakura, Alphen aan den Rijn, Netherlands).

Frozen sections were fixed in 4% paraformaldehyde and analyzed by immunoperoxidase labelling with anti-human nuclei antibody (Millipore, Temecula, CA, USA). Sections were counterstained with haematoxylin. The whole area of each tissue was photographed and digitalized using a Zeiss Axiocam ERc5s camera and Zen 2012 software (Zeiss, Jena, Germany) on a Zeiss Axio Scope.A1 microscope, and the number of human RASF nuclei per air pouch wall area was quantified.

### Statistical analysis

Data were analyzed using GraphPad Prism software v6.0 (GraphPad Software, San Diego, CA, USA). Means were compared by Wilcoxon and Mann-Whitney U-test as appropriate. A p value < 0.05 was considered statistically significant.

## Electronic supplementary material

Supplementary information
