# Supplementary material for: Hif-1α Knockdown Reduces Glycolytic Metabolism and Induces Cell Death of Human Synovial Fibroblasts Under Normoxic Conditions
Source: Sci Rep. 2017 Jun 16;7:3644. doi: 10.1038/s41598-017-03921-4 (PMC5473902; doi:10.1038/s41598-017-03921-4)

HIF-1α KNOCKDOWN REDUCES GLYCOLYTIC METABOLISM AND INDUCES CELL DEATH OF HUMAN SYNOVIAL FIBROBLASTS UNDER NORMOXIC CONDITIONS

Manuel J. Del Rey

Álvaro Valín

Alicia Usategui

Carmen M. García-Herrero

María Sánchez-Aragó

José M. Cuezva

María Galindo

Beatriz Bravo

Juan D. Cañete

Francisco J. Blanco

Gabriel Criado

José L. Pablos

Table S1. Top up- and down-regulated proteins involved in energy metabolism, apoptotic and cell proliferation pathways with fold change siHIF-1α (HIF) versus non-silencing (CTRL) siRNA SF less than 0.6 for down-regulated or greater than 2 for up-regulated proteins (in **bold** letter) in at least one of the two comparisons performed.

Down-regulated

Accession#	Name (symbol)	Peptides (95%)	HIF1:CTRL1 (Pval)	HIF2:CTRL2 (Pval)	Biological process (GO-Gene Ontology)
P09211	Glutathione S-transferase P (GSTP1)	11	0.70 (0.71)	**0.43** (0.42)	Glutathione metabolic process (GO:0006749) Negative regulation of apoptotic process (GO:0043066)
P24752	Acetyl-CoA acetyltransferase (ACAT1)	2	**0.59** (0.41)	1.01 (0.97)	Acetyl-CoA biosynthetic process (GO:0006085)
P04406	Glyceraldehyde-3-phosphate dehydrogenase (GAPDH)	18	**0.58** (0.66)	0.76 (0.31)	Canonical glycolysis (GO:0061621)
P06733	Alpha-enolase (ENO1)	39	**0.55** (<u>0.01</u>)	**0.41** (0.18)	Canonical glycolysis (GO:0061621)
P60174	Triosephosphate isomerase (TPI1)	18	**0.47** (<u>0.007</u>)	0.80 (0.42)	Canonical glycolysis (GO:0061621)
P16070	CD44 antigen (CD44)	3	**0.60** (0.52)	0.64 (0.62)	Negative regulation of apoptotic process (GO:0043066)
P14174	Macrophage migration inhibitory factor (MIF)	3	**0.56** (0.23)	0.91 (0.90)	Negative regulation of apoptotic process (GO:0043066)
P09936	Ubiquitin carboxyl-terminal hydrolase isozyme L1 (UCHL1)	3	**0.54** (0.18)	0.95 (0.92)	Cell proliferation (GO:0008283)
P05556	Integrin beta-1 (ITGB1)	2	**0.53** (0.38)	0.95 (0.80)	Positive regulation of apoptotic process (GO:0043065)

Up-regulated

Accession#	Name (symbol)	Peptides (95%)	HIF1:CTRL1 (Pval)	HIF2:CTRL2 (Pval)	Biological process (GO-Gene Ontology)
P40926	Malate dehydrogenase (MDH2)	6	**2.03** (0.14)	1.74 (0.14)	Gluconeogenesis (GO:0006094)
Q16822	Phosphoenolpyruvate carboxykinase (PCK2)	1	**2.15** (0.24)	1.26 (0.75)	Gluconeogenesis (GO:0006094)
Q9NQC3	Reticulon-4 (RTN4)	3	**3.66** (0.31)	**2.21** (0.41)	Apoptotic process (GO:0006915)
P00441	Superoxide dismutase (SOD1)	1	**2.91** (0.22)	1.51 (0.48)	Positive regulation of apoptotic process (GO:0043065)
P27797	Calreticulin (CALR)	16	**2.61** (0.47)	1.45 (0.59)	Regulation of apoptotic process (GO:0042981)
P35580	Myosin-10 (MYH10)	3	**2.21** (0.29)	1.71 (0.58)	Cell proliferation (GO:0008283)

Table S2. iTRAQ analysis of protein extracts from HIF-1α and control silenced SF

N	Unused	Total	% Cov	Accession #	Name	Peptides (95%)	HIF1:CTRL1 115:114	HIF1:CTRL1 Pval	HIF2:CTRL2 117:116	HIF2:CTRL2 Pval
1	155,37	155,37	51,2	sp|Q09666|AHNK_HUMAN	Neuroblast differentiation-associated protein	101	2,0701	0,0018	1,8197	0
2	112,64	112,64	88,4	sp|P08670|VIME_HUMAN	Vimentin	212	1,7865	0,9426	1,0666	0,8417
3	88,03	88,03	40	sp|P21333|FLNA_HUMAN	Filamin-A	64	1,4454	0,0659	1,7865	0,3605
4	72,92	72,92	43,3	sp|P35579|MYH9_HUMAN	Myosin-9	53	1,6749	0,0516	0,9204	0,1025
5	70,19	70,19	39,7	sp|Q15149|PLEC_HUMAN	Plectin	39	1,5417	0,0384	0,9204	0,7883
6	58,56	58,56	74,7	sp|P60709|ACTB_HUMAN	Actin, cytoplasmic 1	82				
7	56,11	56,11	70,2	sp|P07355|ANXA2_HUMAN	Annexin A2	45	2,9648	0,1124	0,7244	0,0411
8	54,96	54,96	58,1	sp|P11021|GRP78_HUMAN	78 kDa glucose-regulated protein	47	0,8166	0,3279	1,1169	0,4235
9	46,01	46,01	71,2	sp|P06733|ENOA_HUMAN	Alpha-enolase 1	39	0,5495	0,0132	0,413	0,1821
10	41,96	41,96	61	sp|P02545|LMNA_HUMAN	Prelamin-A/C	29	0,9376	0,5736	1,5849	0,0894
11	40,64	40,64	78	sp|P14618|KPYM_HUMAN	Pyruvate kinase isozymes M1/M2	31	1,3677	0,2733	0,7379	0,9332
12	40,5	40,5	56,9	sp|P07237|PDIA1_HUMAN	Protein disulfide-isomerase	29	1,028	0,7307	1,028	0,6669
13	38,68	38,68	46,3	sp|O43707|ACTN4_HUMAN	Alpha-actinin-4	27	1,3428	0,9272	1,3552	0,5232
14	36,14	36,14	55,2	sp|P07437|TBB5_HUMAN	Tubulin beta chain	46	0,7943	0,6794	0,8551	0,8194
15	34,13	36,17	48,3	sp|P11142|HSP7C_HUMAN	Heat shock cognate 71 kDa protein	32	0,912	0,6795	0,9462	0,8022
16	32,62	32,62	27,5	sp|Q9Y490|TLN1_HUMAN	Talin-1	19	1	0,62	0,9908	0,963
17	32	32	50,8	sp|Q71U36|TBA1A_HUMAN	Tubulin alpha-1A chain	37				
18	30,07	30,07	58,1	sp|P08758|ANXA5_HUMAN	Annexin A5	28	2,5119	0,5175	0,6668	0,0651
19	30,02	30,02	75,4	sp|P04083|ANXA1_HUMAN	Annexin A1	16	0,6368	0,688	1,2706	0,8277
20	26,11	26,11	47,5	sp|P30101|PDIA3_HUMAN	Protein disulfide-isomerase A3	19	1,028	0,2575	0,929	0,1175
21	25,43	25,43	39	sp|P14625|ENPL_HUMAN	Endoplasmin	18	0,912	0,1637	0,9817	0,3521
22	23,28	23,28	48,3	sp|Q16555|DPYL2_HUMAN	Dihydropyrimidinase-related protein 2	16	0,9376	0,6596	0,9817	0,966
23	21,75	21,75	69,5	sp|P60174|TPIS_HUMAN	Triosephosphate isomerase	18	0,4699	0,0067	0,8017	0,4214
24	21,24	23,29	34,5	sp|P08238|HS90B_HUMAN	Heat shock protein HSP 90-beta	12	1,1803	0,2146	1,0186	0,634
25	21,03	25,05	18,2	sp|Q14315|FLNC_HUMAN	Filamin-C	13	1	0,8315	0,9727	0,9111
26	20,5	20,5	27,1	sp|P27797|CALR_HUMAN	Calreticulin	16	2,6062	0,4754	1,4454	0,5918
27	20,46	20,46	58,1	sp|P67936|TPM4_HUMAN	Tropomyosin alpha-4 chain	15	0,8091	0,8756	1,2023	0,573
28	20,02	20,02	42,6	sp|Q05682|CALD1_HUMAN	Caldesmon	14	2,0324	0,079	1,7378	0,1481
29	20	20	75,2	sp|P62937|PPIA_HUMAN	Peptidyl-prolyl cis-trans isomerase A	18	0,4786	0,0692	1,1695	0,4635
30	19,89	19,89	34,5	sp|P02452|CO1A1_HUMAN	Collagen alpha-1(I) chain	10	0,912	0,5911	1	0,9363
31	19,35	19,35	33,1	sp|P26038|MOES_HUMAN	Moesin	12	1,6904	0,1876	1,8197	0,0345
32	18,21	18,21	72,6	sp|Q01995|TAGL_HUMAN	Transgelin	14	3,1915	0,0203	0,7943	0,9641
33	16,7	16,7	36,6	sp|O60701|UGDH_HUMAN	UDP-glucose 6-dehydrogenase	11	1,0471	0,6143	1	0,9867
34	16,41	16,41	40,8	sp|P13489|RINI_HUMAN	Ribonuclease inhibitor	11	0,673	0,5695	0,8872	0,9749
35	16,18	16,18	68,1	sp|P04406|G3P_HUMAN	Glyceraldehyde-3-phosphate dehydrogenase	18	0,5808	0,6594	0,7586	0,3079
36	16,07	16,07	56,6	sp|P04075|ALDOA_HUMAN	Fructose-bisphosphate aldolase A	19	0,9204	0,8686	0,8872	0,455
37	16,01	16,01	77	sp|P09382|LEG1_HUMAN	Galectin-1	18	1,9055	0,7689	0,3981	0,5486
38	16	16	50,8	sp|Q06830|PRDX1_HUMAN	Peroxiredoxin-1	12	0,9727	0,4027	1,0666	0,0789
39	15,89	15,89	54,8	sp|P37802|TAGL2_HUMAN	Transgelin-2	14	0,955	0,7533	0,863	0,3483
40	15,66	15,66	43,7	sp|P00558|PGK1_HUMAN	Phosphoglycerate kinase 1	10	0,871	0,9281	0,9204	0,7927
41	15,26	15,26	20,5	sp|P12111|CO6A3_HUMAN	Collagen alpha-3(VI) chain	10	1,1912	0,3779	2,421	0,0894
42	15,26	15,26	19,7	sp|P13639|EF2_HUMAN	Elongation factor 2	9	1,1169	0,245	1,0186	0,4864
43	14,73	14,73	29,6	sp|P38646|GRP75_HUMAN	Stress-70 protein, mitochondrial	9	0,9727	0,9621	0,9727	0,6494

**44**	14,72	14,72	41,5	sp|Q07065|CKAP4_HUMAN	Cytoskeleton-associated protein 4	10	0,5754	0,1222	1,0666	0,8589
**45**	14,01	14,01	56,3	sp|P62805|H4_HUMAN	Histone H4	11	6,9823	0,0954	1,2823	0,1167
**46**	14	14	31,2	sp|P68104|EF1A1_HUMAN	Elongation factor 1-alpha 1	13	1,0666	0,6019	0,929	0,3417
**47**	14	14	48,6	sp|P09211|GSTP1_HUMAN	Glutathione S-transferase P	11	0,6982	0,7137	0,4325	0,4249
**48**	13,74	13,74	58,6	sp|P07737|PROF1_HUMAN	Profilin-1	15	1,4997	0,576	0,5012	0,115
**49**	13,64	13,64	38,8	sp|P06576|ATPB_HUMAN	ATP synthase subunit beta, mitochondrial	10	1,0093	0,7514	0,9638	0,5535
**50**	13,52	13,52	19,1	sp|Q00610|CLH1_HUMAN	Clathrin heavy chain 1	7	0,6138	0,3919	1,5996	0,3161
**51**	13,27	13,27	31	sp|Q8NBS9|TXND5_HUMAN	Thioredoxin domain-containing protein 5	7	0,863	0,1087	1	0,6435
**52**	13,18	13,18	53,5	sp|O60664|PLIN3_HUMAN	Perilipin-3	8	0,863	0,5202	0,7311	0,1116
**53**	13,01	13,01	36,1	sp|P50454|SERPH_HUMAN	Serpin H1	11	1,1376	0,7604	0,6368	0,176
**54**	12,97	12,97	27,3	sp|P55072|TERA_HUMAN	Transitional endoplasmic reticulum ATPase	9	0,8241	0,2226	1,0965	0,4224
**55**	12,56	12,56	59,7	sp|P23284|PPIB_HUMAN	Peptidyl-prolyl cis-trans isomerase B	10	0,955	0,4948	0,7244	0,3019
**56**	12,53	12,53	42,5	sp|P63104|1433Z_HUMAN	14-3-3 protein zeta/delta	6	1,0471	0,7907	1,3062	0,1493
**57**	12,27	12,27	65,6	sp|P60660|MYL6_HUMAN	Myosin light polypeptide 6	11	1,0375	0,5057	0,9727	0,5138
**58**	12,21	12,21	27,8	sp|Q96AY3|FKB10_HUMAN	Peptidyl-prolyl cis-trans isomerase	9	1,0568	0,6134	0,9462	0,7204
**59**	11,58	11,58	34,3	sp|P21796|VDAC1_HUMAN	Voltage-dependent anion-selective channel protein 1	6	1	0,9148	1,0568	0,4678
**60**	11,52	11,52	26,6	sp|Q15084|PDIA6_HUMAN	Protein disulfide-isomerase A6	11	0,863	0,9677	0,492	0,7672
**61**	11,05	11,05	39,5	sp|Q6NZI2|PTRF_HUMAN	Polymerase I and transcript release factor	12	1,4723	0,1085	1,4588	0,4775
**62**	10,97	10,97	34,5	sp|P07858|CATB_HUMAN	Cathepsin B	7	1,5996	0,2956	1,5849	0,8625
**63**	10,9	30,08	39,6	sp|P12814|ACTN1_HUMAN	Alpha-actinin-1	18	1,0666	0,6268	1,3062	0,1133
**64**	10,61	10,61	24,8	sp|P07339|CATD_HUMAN	Cathepsin D	9	0,9727	0,6254	0,8872	0,39
**65**	10,43	10,43	36,9	sp|O00299|CLIC1_HUMAN	Chloride intracellular channel protein 1	7	0,7244	0,4902	0,6252	0,2544
**66**	10,38	10,38	27	sp|P08133|ANXA6_HUMAN	Annexin A6	5	2,2699	0,0918	0,6855	0,5552
**67**	10,01	10,01	36,7	sp|P40926|MDHM_HUMAN	Malate dehydrogenase, mitochondrial	6	2,0324	0,1385	1,7378	0,1391
**68**	10	10	17,7	sp|Q9P2E9|RRBP1_HUMAN	Ribosome-binding protein 1	8	1,0471	0,2979	0,8872	0,6099
**69**	10	10	13,6	sp|P22314|UBA1_HUMAN	Ubiquitin-like modifier-activating enzyme 1	8	0,955	0,49	0,9908	0,8752
**70**	10	10	46	sp|Q8N257|H2B3B_HUMAN	Histone H2B type 3-B	15	1,5417	0,4632	1,1695	0,7398
**71**	10	10	44	sp|P23528|COF1_HUMAN	Cofilin-1	8	0,955	0,7759	0,9638	0,5059
**72**	10	10	49,5	sp|P60903|S10AA_HUMAN	Protein S100-A10	9	1,1169	0,3856	1,2589	0,0415
**73**	10	10	45,6	sp|P62158|CALM_HUMAN	Calmodulin	6	2,3988	0,2661	1,5704	0,4475
**74**	9,77	9,77	16,6	sp|Q99715|COCA1_HUMAN	Collagen alpha-1(XII) chain	7	0,9204	0,6066	0,7656	0,4514
**75**	9,6	9,6	23,8	sp|P18206|VINC_HUMAN	Vinculin	6	0,6138	0,2315	0,3373	0,0043
**76**	9,39	9,39	39,9	sp|Q15365|PCBP1_HUMAN	Poly(rC)-binding protein 1	5				
**77**	8,78	8,78	36,5	sp|P25705|ATPA_HUMAN	ATP synthase subunit alpha, mitochondrial	9	0,9376	0,982	1,0471	0,4299
**78**	8,71	8,71	24,7	sp|P08123|CO1A2_HUMAN	Collagen alpha-2(I) chain	7	0,9376	0,678	0,9638	0,868
**79**	8,54	8,54	24,6	sp|P10809|CH60_HUMAN	60 kDa heat shock protein, mitochondrial	9	0,8872	0,3965	0,955	0,289
**80**	8,37	8,37	17,4	sp|Q13813|SPTA2_HUMAN	Spectrin alpha chain, brain	4	1,6904	0,1379	1,1376	0,6843
**81**	8,34	18,54	43,3	sp|Q9BUF5|TBB6_HUMAN	Tubulin beta-6 chain	21	1,0471	0,9049	1,0568	0,8797
**82**	8,31	8,31	28,4	sp|P26641|EF1G_HUMAN	Elongation factor 1-gamma	6	0,863	0,3069	1	0,8826
**83**	8,16	8,16	23,2	sp|Q99536|VAT1_HUMAN	Synaptic vesicle membrane protein VAT-1 homolog	6	1,0471	0,8567	1,1482	0,1724
**84**	8,08	8,08	20,6	sp|P29401|TKT_HUMAN	Transketolase	7	0,9638	0,4713	0,9727	0,8408
**85**	8,05	8,05	21,1	sp|P06396|GELS_HUMAN	Gelsolin	5	0,929	0,645	1,0093	0,8838
**86**	8	8	14,9	sp|P27824|CALX_HUMAN	Calnexin	5	1,1376	0,531	1,0093	0,9137
**87**	8	8	22,6	sp|P00338|LDHA_HUMAN	L-lactate dehydrogenase A chain	7	1,1169	0,4881	0,9638	0,4397
**88**	8	8	15,3	sp|P21589|5NTD_HUMAN	5'-nucleotidase	4	1,0093	0,5166	0,912	0,4527
**89**	8	8	34,4	sp|Q71UI9|H2AV_HUMAN	Histone H2A.V	5	0,8166	0,6261	0,9638	0,9265
**90**	8	8	12,6	sp|P07602|SAP_HUMAN	Proactivator polypeptide	4	1,4723	0,5931	1,5996	0,3552
**91**	7,92	7,92	39,8	sp|P18669|PGAM1_HUMAN	Phosphoglycerate mutase 1	5	0,7447	0,706	0,6427	0,3957

**92**	7,59	12,22	21,2	sp|O75369|FLNB_HUMAN	Filamin-B	10	0,9727	0,7566	1,0765	0,3795
**93**	7,15	7,15	40,4	sp|P62277|RS13_HUMAN	40S ribosomal protein S13	4	1,028	0,5472	0,9908	0,5014
**94**	7,05	7,05	18	sp|P31943|HNRH1_HUMAN	Heterogeneous nuclear ribonucleoprotein H	8	1,0471	0,1159	0,9462	0,1648
**95**	6,68	6,68	20	sp|O43852|CALU_HUMAN	Calumenin	5	1,0375	0,7795	0,912	0,7928
**96**	6,58	6,58	28,1	sp|O60506|HNRPQ_HUMAN	Heterogeneous nuclear ribonucleoprotein Q	3	1,1482	0,371	1,0965	0,6884
**97**	6,47	6,47	22	sp|O43175|SERA_HUMAN	D-3-phosphoglycerate dehydrogenase	3	1,3305	0,325	0,929	0,8148
**98**	6,46	6,46	40,5	sp|P04792|HSPB1_HUMAN	Heat shock protein beta-1	8	0,5808	0,2678	0,6918	0,763
**99**	6,31	6,31	18,6	sp|Q96D15|RCN3_HUMAN	Reticulocalbin-3	4	2,0701	0,2541	1,9953	0,1972
**100**	6,22	6,22	17,3	sp|P15144|AMPN_HUMAN	Aminopeptidase N	5	2,421	0,0315	0,8872	0,8354
**101**	6,11	6,11	42,9	sp|P06748|NPM_HUMAN	Nucleophosmin	5	0,7516	0,4767	1,1482	0,8912
**102**	6,11	6,11	52,2	sp|P84243|H33_HUMAN	Histone H3.3	3	1,5996	0,1649	1,2359	0,4377
**103**	6,1	6,1	32	sp|P60842|IF4A1_HUMAN	Eukaryotic initiation factor 4A-I	4	1,2023	0,3255	0,8395	0,5707
**104**	6,1	6,1	27,6	sp|P12277|KCRB_HUMAN	Creatine kinase B-type	5	0,8017	0,2161	0,879	0,7428
**105**	6,09	6,09	22	sp|P17931|LEG3_HUMAN	Galectin-3	4	1,2589	0,5069	0,955	0,7175
**106**	6,02	6,02	35,8	sp|P30050|RL12_HUMAN	60S ribosomal protein L12	4	1,0568	0,7126	0,9638	0,9533
**107**	6,01	6,01	43,7	sp|P50914|RL14_HUMAN	60S ribosomal protein L14	5	0,8318	0,5736	1,0765	0,6994
**108**	6	34,14	55,1	sp|P68371|TBB2C_HUMAN	Tubulin beta-2C chain	43	0,9727	0,8891	1,0965	0,497
**109**	6	7,32	22,7	sp|Q15366|PCBP2_HUMAN	Poly(rC)-binding protein 2	5	0,8017	0,7581	0,7586	0,2639
**110**	6	6	8,3	sp|Q9NQC3|RTN4_HUMAN	Reticulon-4	3	3,6644	0,3109	2,208	0,411
**111**	6	6	28,9	sp|P62701|RS4X_HUMAN	40S ribosomal protein S4, X isoform	4	0,9204	0,8628	0,9376	0,907
**112**	6	6	28,9	sp|P46782|RS5_HUMAN	40S ribosomal protein S5	4	0,7798	0,5913	1,1803	0,6654
**113**	6	6	56,2	sp|P31949|S10AB_HUMAN	Protein S100-A11	7	0,7047	0,3717	0,4786	0,1
**114**	6	6	39,1	sp|P10599|THIO_HUMAN	Thioredoxin	5	0,7311	0,2533	1,0093	0,5135
**115**	5,89	5,89	29,9	sp|P00387|NB5R3_HUMAN	NADH-cytochrome b5 reductase 3	3	1,1272	0,5231	0,673	0,4758
**116**	5,61	5,61	31,5	sp|P60981|DEST_HUMAN	Destrin	4	0,9908	0,4928	0,2938	0,1546
**117**	5,55	5,56	42,7	sp|P16403|H12_HUMAN	Histone H1.2	4	0,6194	0,3458	1,3428	0,4358
**118**	5,5	5,5	19,7	sp|P19338|NUCL_HUMAN	Nucleolin	3	0,7727	0,9454	1,0186	0,9359
**119**	5,37	5,37	32,9	sp|P15531|NDKA_HUMAN	Nucleoside diphosphate kinase A	5	0,9376	0,7556	1,028	0,7597
**120**	5,32	5,32	34,5	sp|P09936|UCHL1_HUMAN	Ubiquitin carboxyl-terminal hydrolase isozyme L1	3	0,5445	0,1814	0,9462	0,9197
**121**	5,22	7,52	26,7	sp|P08107|HSP71_HUMAN	Heat shock 70 kDa protein 1A/1B	5	1,028	0,8787	1,028	0,668
**122**	5,16	5,16	15,1	sp|O15460|P4HA2_HUMAN	Prolyl 4-hydroxylase subunit alpha-2	3	0,8872	0,657	0,7112	0,2396
**123**	4,82	4,82	22,8	sp|Q96AG4|LRC59_HUMAN	Leucine-rich repeat-containing protein 59	3	0,9204	0,8496	0,9376	0,8893
**124**	4,77	4,77	17,9	sp|Q01082|SPTB2_HUMAN	Spectrin beta chain, brain 1	2	5,5976	0,0892	1,0965	0,8348
**125**	4,68	4,68	12,9	sp|Q9NZN4|EHD2_HUMAN	EH domain-containing protein 2	3	1,2359	0,1709	0,955	0,7136
**126**	4,56	4,56	19,8	sp|P26447|S10A4_HUMAN	Protein S100-A4	2	0,6668	0,3238	0,2992	0,1146
**127**	4,46	4,46	68,2	sp|P63313|TYB10_HUMAN	Thymosin beta-10	3	1,1588	0,758	1,1695	0,751
**128**	4,42	4,42	21,4	sp|P41250|SYG_HUMAN	Glycyl-tRNA synthetase	2	0,7727	0,3718	0,863	0,1706
**129**	4,36	4,36	18,1	sp|P42224|STAT1_HUMAN	Signal transducer and activator of transcription 1-alpha/beta	3	1,2023	0,4909	1,0186	0,9723
**130**	4,27	4,27	19,9	sp|P00367|DHE3_HUMAN	Glutamate dehydrogenase 1, mitochondrial	3	0,9376	0,9007	1,1066	0,7299
**131**	4,09	4,09	34,9	sp|P29692|EF1D_HUMAN	Elongation factor 1-delta	2	1,0186	0,8506	1	0,9914
**132**	4,09	4,09	13,8	sp|Q96HC4|PDLI5_HUMAN	PDZ and LIM domain protein 5	2	1,2246	0,6895	1,1272	0,8041
**133**	4,07	4,07	29,2	sp|P19105|ML12A_HUMAN	Myosin regulatory light chain 12A	5	0,9817	0,9612	0,8551	0,6557
**134**	4,02	4,02	14	sp|P05023|AT1A1_HUMAN	Sodium/potassium-transporting ATPase subunit alpha-1	2	0,912	0,4444	1,0186	0,726
**135**	4,02	4,02	18,4	sp|P40121|CAPG_HUMAN	Macrophage-capping protein	3	0,871	0,5121	0,9908	0,9809
**136**	4,01	4,01	13,9	sp|O95302|FKBP9_HUMAN	Peptidyl-prolyl cis-trans isomerase	2	0,9908	0,9647	1,1272	0,3699
**137**	4,01	4,01	19,6	sp|P61158|ARP3_HUMAN	Actin-related protein 3	2	0,787	0,6648	0,4246	0,2769
**138**	4,01	4,01	32,2	sp|O95336|6PGL_HUMAN	6-phosphogluconolactonase	2	0,7447	0,541	0,787	0,688
**139**	4	38,2	61	sp|P68032|ACTC_HUMAN	Actin, alpha cardiac muscle 1	43	1,0864	0,8469	0,8551	0,7702

**140**	4	32	50,8	sp|P68363|TBA1B_HUMAN	Tubulin alpha-1B chain	37	0,8954	0,7982	0,9462	0,8681
**141**	4	10	46,8	sp|Q99879|H2B1M_HUMAN	Histone H2B type 1-M	16	0,9638	0,9598	1,0375	0,9746
**142**	4	4	22,2	sp|P49368|TCPG_HUMAN	T-complex protein 1 subunit gamma	2	0,8872	0,7612	0,8091	0,5578
**143**	4	4	30,8	sp|Q15019|SEPT2_HUMAN	Septin-2	2	0,912	0,4424	1,1695	0,1464
**144**	4	4	34,5	sp|P62753|RS6_HUMAN	40S ribosomal protein S6	3	1,4997	0,548	1,2023	0,661
**145**	4	4	36	sp|P61247|RS3A_HUMAN	40S ribosomal protein S3a	4	0,7798	0,5881	0,929	0,8636
**146**	4	4	15,6	sp|P52272|HNRPM_HUMAN	Heterogeneous nuclear ribonucleoprotein M	2	1,4454	0,5102	0,8472	0,7612
**147**	4	4	53,4	sp|P62847|RS24_HUMAN	40S ribosomal protein S24	2	0,8872	0,821	1,556	0,4549
**148**	4	4	18	sp|P50395|GDIB_HUMAN	Rab GDP dissociation inhibitor beta	2	1,7378	0,3652	0,7447	0,5768
**149**	4	4	33,6	sp|P08865|RSSA_HUMAN	40S ribosomal protein SA	2	1,2246	0,6618	1,0375	0,8035
**150**	4	4	20,9	sp|P07910|HNRPC_HUMAN	Heterogeneous nuclear ribonucleoproteins C1/C2	2	0,8954	0,855	1,0965	0,7936
**151**	4	4	19,5	sp|P07195|LDHB_HUMAN	L-lactate dehydrogenase B chain	2	1,2589	0,1427	1,1588	0,1791
**152**	4	4	20,7	sp|Q14847|LASP1_HUMAN	LIM and SH3 domain protein 1	2	1,1376	0,8213	1,0765	0,8335
**153**	4	4	14,1	sp|Q01518|CAP1_HUMAN	Adenylyl cyclase-associated protein 1	3	0,4613	0,3025	0,7656	0,6315
**154**	4	4	13,5	sp|P78371|TCPB_HUMAN	T-complex protein 1 subunit beta	2	1,1588	0,687	0,4246	0,2067
**155**	4	4	38,8	sp|P46778|RL21_HUMAN	60S ribosomal protein L21	2	0,9036	0,8482	1,5417	0,462
**156**	4	4	21,1	sp|P45880|VDAC2_HUMAN	Voltage-dependent anion-selective channel protein 2	2	1,0471	0,811	0,7943	0,4923
**157**	4	4	13,4	sp|P15289|ARSA_HUMAN	Arylsulfatase A	2	1	0,9079	1,6596	0,2698
**158**	4	4	20,1	sp|P09525|ANXA4_HUMAN	Annexin A4	2	1,1272	0,6263	0,9036	0,69
**159**	4	4	19,2	sp|P04439|1A03_HUMAN	HLA class I histocompatibility antigen, A-3 alpha chain	2	0,8954	0,7544	0,8318	0,8517
**160**	4	4	28,6	sp|P62906|RL10A_HUMAN	60S ribosomal protein L10a	2	1,0765	0,3483	0,9727	0,8748
**161**	4	4	32,9	sp|P62829|RL23_HUMAN	60S ribosomal protein L23	3	0,8472	0,9433	19,2309	0,3582
**162**	4	4	18,1	sp|P52565|GDIR1_HUMAN	Rho GDP-dissociation inhibitor 1	3	1,2706	0,4101	1,1272	0,6885
**163**	4	4	22,1	sp|P04179|SODM_HUMAN	Superoxide dismutase [Mn], mitochondrial	3	1,0568	0,3701	1,028	0,6428
**164**	4	4	27,9	sp|Q6IS14|IF5AL_HUMAN	Eukaryotic translation initiation factor 5A-1-like	3	0,7798	0,6339	1,5417	0,4392
**165**	4	4	42	sp|P60866|RS20_HUMAN	40S ribosomal protein S20	2	1,0375	0,9225	0,7178	0,5637
**166**	4	4	11,9	sp|P43307|SSRA_HUMAN	Translocon-associated protein subunit alpha	2	1,977	0,2812	0,8318	0,9157
**167**	4	4	24,8	sp|P30044|PRDX5_HUMAN	Peroxiredoxin-5, mitochondrial	3	1,1803	0,9715	1,3305	0,2742
**168**	4	4	29,7	sp|P13987|CD59_HUMAN	CD59 glycoprotein	4	0,9817	0,9829	1,0568	0,8921
**169**	4	4	49	sp|P04080|CYTB_HUMAN	OS=Homo sapiens GN=CSTB PE=1 SV=2	2	1,0765	0,87	1,0186	0,9541
**170**	4	4	20,2	sp|O75915|PRAF3_HUMAN	PRA1 family protein 3	2	1,0765	0,6079	0,8017	0,62
**171**	4	4	25	sp|P62942|FKB1A_HUMAN	Peptidyl-prolyl cis-trans isomerase	2	1,6749	0,5783	1,2823	0,7255
**172**	4	4	9,8	sp|P24534|EF1B_HUMAN	Elongation factor 1-beta	3	1,0093	0,915	1	0,979
**173**	4	4	17,4	sp|P14174|MIF_HUMAN	Macrophage migration inhibitory factor	3	0,5649	0,2341	0,912	0,9018
**174**	4	4	13,7	sp|P02511|CRYAB_HUMAN	Alpha-crystallin B chain	2	1,9231	0,5287	1,7378	0,5803
**175**	3,8	3,8	19	sp|P50995|ANX11_HUMAN	Annexin A11	2	1,2706	0,6174	1,1376	0,693
**176**	3,7	3,7	10,6	sp|P14314|GLU2B_HUMAN	Glucosidase 2 subunit beta	2	1,0568	0,8792	1,0765	0,9768
**177**	3,65	3,65	15,7	sp|P30040|ERP29_HUMAN	Endoplasmic reticulum resident protein 29	2	1,0568	0,6679	0,9036	0,8555
**178**	3,59	3,59	27,4	sp|P84103|SRSF3_HUMAN	Serine/arginine-rich splicing factor 3	2	0,6918	0,4578	0,863	0,7341
**179**	3,54	3,54	57	sp|P62987|RL40_HUMAN	Ubiquitin-60S ribosomal protein L40	4	0,673	0,4924	1,803	0,3368
**180**	3,18	3,18	26,7	sp|O95865|DDAH2_HUMAN	N(G),N(G)-dimethylarginine dimethylaminohydrolase 2	2	0,9376	0,8607	0,8241	0,5806
**181**	3,11	3,11	9,7	sp|Q14974|IMB1_HUMAN	Importin subunit beta-1	2	0,7112	0,719	0,5012	0,3021
**182**	3,09	3,09	41,2	sp|P26373|RL13_HUMAN	60S ribosomal protein L13	3	0,955	0,981	1,2589	0,4265
**183**	3,02	3,02	44,4	sp|P06703|S10A6_HUMAN	Protein S100-A6	2	1,4322	0,2872	0,7379	0,3247
**184**	3	12,33	20,8	sp|P07900|HS90A_HUMAN	Heat shock protein HSP 90-alpha	7	5,5976	0,0533	0,8241	0,3186
**185**	2,93	4,08	26,6	sp|P16401|H15_HUMAN	Histone H1.5	2				
**186**	2,83	2,83	19,5	sp|P49588|SYAC_HUMAN	Alanyl-tRNA synthetase, cytoplasmic	2	1,1588	0,3861	0,955	0,7526
**187**	2,79	2,79	20,5	sp|P40939|ECHA_HUMAN	Trifunctional enzyme subunit alpha, mitochondrial	3	1,0568	0,7164	0,787	0,2251

**188**	2,73	2,73	27,5	sp|Q9Y2B0|CNPY2_HUMAN	Protein canopy homolog 2	2	1,0093	0,9889	0,9036	0,9567
**189**	2,7	2,7	14	sp|Q8IVF2|AHNK2_HUMAN	Protein AHNAK2	2	1,1169	0,1847	1,4454	0,0954
**190**	2,63	2,63	27,5	sp|P62857|RS28_HUMAN	40S ribosomal protein S28	2	6,4863	0,2644	1,4191	0,3935
**191**	2,61	2,61	10,9	sp|P16070|CD44_HUMAN	CD44 antigen	3	0,597	0,5247	0,6427	0,6169
**192**	2,6	2,6	10,2	sp|P46940|IQGA1_HUMAN	Ras GTPase-activating-like protein IQGAP1	2	1,1376	0,6233	2,2284	0,2595
**193**	2,58	2,58	12,9	sp|Q92841|DDX17_HUMAN	Probable ATP-dependent RNA helicase DDX17	2	1,1272	0,6416	1,0765	0,7435
**194**	2,48	5,43	24,3	sp|P08729|K2C7_HUMAN	Keratin, type II cytoskeletal 7	4	1,1066	0,8232	1,4454	0,5146
**195**	2,47	2,47	10,4	sp|Q02878|RL6_HUMAN	60S ribosomal protein L6	2	0,9376	0,9038	0,9036	0,7875
**196**	2,41	2,41	19,4	sp|Q14764|MVP_HUMAN	Major vault protein	2	1,1912	0,7159	1,2134	0,698
**197**	2,38	2,38	6,9	sp|P34932|HSP74_HUMAN	Heat shock 70 kDa protein 4	3	1,0965	0,8783	1	0,7915
**198**	2,34	2,34	11,8	sp|P55209|NP1L1_HUMAN	Nucleosome assembly protein 1-like 1	2	0,9376	0,8716	1,1066	0,776
**199**	2,33	2,33	39,2	sp|P61604|CH10_HUMAN	10 kDa heat shock protein, mitochondrial	1	1,0471	0,3199	1,0093	0,7067
**200**	2,31	2,31	9,8	sp|Q02818|NUCB1_HUMAN	Nucleobindin-1	1	0,929	0,6602	0,6607	0,288
**201**	2,29	2,29	42,1	sp|P62269|RS18_HUMAN	40S ribosomal protein S18	1	2,7542	0,2262	1,9055	0,3385
**202**	2,28	2,28	31,5	sp|P61978|HNRPK_HUMAN	Heterogeneous nuclear ribonucleoprotein K	1	1,1803	0,0333	0,6792	0,0795
**203**	2,2	2,2	13,5	sp|P04843|RPN1_HUMAN	Dolichyl-diphosphooligosaccharide--protein glycosyltransferase subunit 1	2	0,7178	0,4118	1,0864	0,9964
**204**	2,17	2,17	15,8	sp|O75396|SC22B_HUMAN	Vesicle-trafficking protein SEC22b	1	1,0864	0,9228	1,0765	0,8572
**205**	2,14	2,14	23,7	sp|P30041|PRDX6_HUMAN	Peroxiredoxin-6	1	1,1066	0,8333	1,0093	0,9632
**206**	2,11	6,22	63,1	sp|Q6FI13|H2A2A_HUMAN	Histone H2A type 2-A	9	0,8318	0,7056	0,871	0,7537
**207**	2,11	2,11	9,7	sp|P53396|ACLY_HUMAN	ATP-citrate synthase	1	1,1588	0,6672	0,863	0,6711
**208**	2,09	2,09	19	sp|Q9ULV4|COR1C_HUMAN	Coronin-1C	1	1	0,931	0,8954	0,5913
**209**	2,06	2,06	18,6	sp|Q02809|PLOD1_HUMAN	Procollagen-lysine,2-oxoglutarate 5-dioxygenase 1	1	0,871	0,5434	0,912	0,6461
**210**	2,06	2,06	13	sp|Q15293|RCN1_HUMAN	Reticulocalbin-1	2	1	0,9789	0,8954	0,8381
**211**	2,05	4	33	sp|P09972|ALDOC_HUMAN	Fructose-bisphosphate aldolase C	3				
**212**	2,04	2,04	32,3	sp|P22626|ROA2_HUMAN	Heterogeneous nuclear ribonucleoproteins A2/B1	1	0,8091	0,6856	0,8395	0,7534
**213**	2,04	2,04	16,4	sp|Q99497|PARK7_HUMAN	Protein DJ-1	1	0,912	0,7449	0,8091	0,7507
**214**	2,03	2,14	22,5	sp|Q8WWI1|LMO7_HUMAN	LIM domain only protein 7	3	1,1169	0,2988	1,1066	0,3559
**215**	2,03	2,03	26,7	sp|P36578|RL4_HUMAN	60S ribosomal protein L4	2	3,4674	0,1927	0,7047	0,5471
**216**	2,03	2,03	9,9	sp|O00159|MYO1C_HUMAN	Myosin-Ic	1				
**217**	2,03	2,03	26,5	sp|O15173|PGRC2_HUMAN	Membrane-associated progesterone receptor component 2	1	1,1376	0,9355	0,879	0,6491
**218**	2,02	2,02	15,7	sp|Q00341|VIGLN_HUMAN	Vigilin	1	1,1803	0,4568	1,0375	0,938
**219**	2,02	2,02	26,8	sp|P83731|RL24_HUMAN	60S ribosomal protein L24	1	1,028	0,9435	1,1066	0,8308
**220**	2,01	2,02	7,6	sp|Q3SY69|AL1L2_HUMAN	Probable 10-formyltetrahydrofolate dehydrogenase ALDH1L2	1	0,7379	0,4316	0,9817	0,7642
**221**	2,01	2,01	12,9	sp|Q00839|HNRPU_HUMAN	Heterogeneous nuclear ribonucleoprotein U	2	0,9204	0,8778	1,4191	0,5282
**222**	2,01	2,01	34,5	sp|P15880|RS2_HUMAN	40S ribosomal protein S2	1	0,929	0,8926	0,9036	0,8476
**223**	2,01	2,01	29,8	sp|P35232|PHB_HUMAN	Prohibitin	2	0,7178	0,5628	1,2706	0,6387
**224**	2,01	2,01	8,9	sp|P23381|SYWC_HUMAN	Tryptophanyl-tRNA synthetase, cytoplasmic	1	0,5395	0,0992	0,5495	0,1662
**225**	2	58,56	74,7	sp|P63261|ACTG_HUMAN	Actin, cytoplasmic 2	89				
**226**	2	30,13	50,8	sp|Q9BVA1|TBB2B_HUMAN	Tubulin beta-2B chain	37	1,4322	0,5333	0,9727	0,963
**227**	2	30	45,7	sp|Q9BQE3|TBA1C_HUMAN	Tubulin alpha-1C chain	32	0,2831	0,193	0,5152	0,3441
**228**	2	7,68	20,1	sp|P15311|EZRI_HUMAN	Ezrin	5	1,5849	0,4451	1,0765	0,8692
**229**	2	5,42	14,2	sp|P35580|MYH10_HUMAN	Myosin-10	3	2,208	0,2881	1,7061	0,3968
**230**	2	4,31	31,4	sp|P27348|1433T_HUMAN	14-3-3 protein theta	2	0,8551	0,7701	1,5276	0,465
**231**	2	2,01	12,6	sp|P13674|P4HA1_HUMAN	Prolyl 4-hydroxylase subunit alpha-1	1	0,929	0,8417	0,5598	0,3668
**232**	2	2	20,2	sp|P33176|KINH_HUMAN	Kinesin-1 heavy chain	2	0,3105	0,2078	0,4246	0,276
**233**	2	2	9,1	sp|P07814|SYEP_HUMAN	Bifunctional aminoacyl-tRNA synthetase	1	1,0375	0,9375	0,9638	0,9417
**234**	2	2	21,6	sp|P04264|K2C1_HUMAN	Keratin, type II cytoskeletal 1	1	0,2655	0,196	0,2992	0,3323

**235**	2	2	10,4	sp|Q8WUM4|PDC6I_HUMAN	Programmed cell death 6-interacting protein	1	0,6026	0,426	3,1915	0,206
**236**	2	2	17,5	sp|P43243|MATR3_HUMAN	Matrin-3	1	1,0186	0,9607	0,8318	0,7181
**237**	2	2	15,1	sp|O95782|AP2A1_HUMAN	AP-2 complex subunit alpha-1	1	1,0568	0,8812	1,7378	0,4243
**238**	2	2	11,2	sp|Q9BSJ8|ESYT1_HUMAN	Extended synaptotagmin-1	1	0,8954	0,582	0,7311	0,7984
**239**	2	2	11,3	sp|Q32P28|P3H1_HUMAN	Prolyl 3-hydroxylase 1	1	1,5996	0,4379	1,6596	0,4137
**240**	2	2	14,9	sp|P10253|LYAG_HUMAN	Lysosomal alpha-glucosidase	1	1	0,9903	1,406	0,5315
**241**	2	2	26,3	sp|P09651|ROA1_HUMAN	Heterogeneous nuclear ribonucleoprotein A1	2	0,9204	0,8793	0,863	0,7807
**242**	2	2	8,8	sp|P05556|ITB1_HUMAN	Integrin beta-1	2	0,5297	0,3793	0,9462	0,8043
**243**	2	2	10	sp|Q9Y678|COPG_HUMAN	Coatomer subunit gamma	1				
**244**	2	2	14,8	sp|Q5JTV8|TOIP1_HUMAN	Torsin-1A-interacting protein 1	1	1,1912	0,6296	1,3552	0,6733
**245**	2	2	13,9	sp|Q13554|KCC2B_HUMAN	Calcium/calmodulin-dependent protein kinase type II subunit beta	1	1,3677	0,4335	1,0471	0,8144
**246**	2	2	13,5	sp|P50990|TCPQ_HUMAN	T-complex protein 1 subunit theta	1	1,0666	0,8912	0,7586	0,6195
**247**	2	2	19	sp|P49411|EFTU_HUMAN	Elongation factor Tu, mitochondrial	1	1,4588	0,503	0,8954	0,8327
**248**	2	2	27,8	sp|P31942|HNRH3_HUMAN	Heterogeneous nuclear ribonucleoprotein H3	2	0,0711	0,3006	0,7943	0,7342
**249**	2	2	15,6	sp|P20073|ANXA7_HUMAN	Annexin A7	1	1,0765	0,839	0,9462	0,7619
**250**	2	2	16,2	sp|P13797|PLST_HUMAN	Plastin-3	1	0,871	0,8013	0,9376	0,9075
**251**	2	2	10,8	sp|O94925|GLSK_HUMAN	Glutaminase kidney isoform, mitochondrial	1	0,673	0,5053	1,1482	0,7667
**252**	2	2	18,9	sp|Q9Y617|SERC_HUMAN	Phosphoserine aminotransferase	1	1,4588	0,5014	0,929	0,8993
**253**	2	2	16,5	sp|Q8N7G0|PO5F2_HUMAN	POU domain, class 5, transcription factor 2	1	0,9462	0,8798	0,879	0,9002
**254**	2	2	10,5	sp|Q14956|GPNMB_HUMAN	Transmembrane glycoprotein NMB	1	1,8535	0,3554	1,6749	0,4088
**255**	2	2	12,8	sp|Q12797|ASPH_HUMAN	Aspartyl/asparaginyl beta-hydroxylase	1	0,9638	0,6922	0,9908	0,5056
**256**	2	2	18,2	sp|P13645|K1C10_HUMAN	Keratin, type I cytoskeletal 10	1	0,1585	0,1343	0,3733	0,2487
**257**	2	2	26,1	sp|P07741|APT_HUMAN	Adenine phosphoribosyltransferase	1	0,9462	0,9202	0,3565	0,2337
**258**	2	2	30,7	sp|O95571|ETHE1_HUMAN	Protein ETHE1, mitochondrial	1	1,0093	0,9527	1	0,776
**259**	2	2	26,4	sp|O75828|CBR3_HUMAN	Carbonyl reductase [NADPH] 3	1	1,1066	0,9279	0,8395	0,5732
**260**	2	2	36,2	sp|Q9Y3U8|RL36_HUMAN	60S ribosomal protein L36	1	1,0864	0,6243	0,8954	0,7626
**261**	2	2	10	sp|Q16822|PCKGM_HUMAN	Phosphoenolpyruvate carboxykinase [GTP], mitochondrial	1	2,1478	0,2433	1,2589	0,7535
**262**	2	2	21,2	sp|Q16698|DECR_HUMAN	2,4-dienoyl-CoA reductase, mitochondrial	1	0,863	0,5875	0,8166	0,8292
**263**	2	2	14,1	sp|P49419|AL7A1_HUMAN	Alpha-aminoadipic semialdehyde dehydrogenase	1	1,556	0,4532	1,028	0,9363
**264**	2	2	16,2	sp|P35998|PRS7_HUMAN	26S protease regulatory subunit 7	1	1,7061	0,3995	1,2474	0,6625
**265**	2	2	17,1	sp|P24752|THIL_HUMAN	Acetyl-CoA acetyltransferase, mitochondrial	2	0,5861	0,4093	1,0093	0,9704
**266**	2	2	26,9	sp|P08134|RHOC_HUMAN	Rho-related GTP-binding protein RhoC	1				
**267**	2	2	14,2	sp|Q96CX2|KCD12_HUMAN	BTB/POZ domain-containing protein KCTD12	1	1	0,6743	0,955	0,3409
**268**	2	2	12,6	sp|Q13418|ILK_HUMAN	Integrin-linked protein kinase	1	1,2023	0,7029	1,0375	0,9275
**269**	2	2	21,6	sp|Q07021|C1QBP_HUMAN	Complement component 1 Q subcomponent-binding protein, mitochondrial	1	0,8241	0,777	99,0832	0,1568
**270**	2	2	9,9	sp|P54578|UBP14_HUMAN	Ubiquitin carboxyl-terminal hydrolase 14	1	0,8318	0,723	0,7943	0,7233
**271**	2	2	9,6	sp|P17813|EGLN_HUMAN	Endoglin	1	2,9648	0,2182	0,7379	0,5865
**272**	2	2	26,2	sp|P16949|STMN1_HUMAN	Stathmin	1	0,6607	0,4537	0,7798	0,5407
**273**	2	2	14,7	sp|P10644|KAP0_HUMAN	cAMP-dependent protein kinase type I-alpha regulatory subunit	1	0,9727	0,9556	0,9817	0,9583
**274**	2	2	23,9	sp|P10301|RRAS_HUMAN	Ras-related protein R-Ras	1	1,4454	0,5079	1,4723	0,498
**275**	2	2	25,3	sp|P00441|SODC_HUMAN	Superoxide dismutase [Cu-Zn]	1	2,9107	0,2229	1,5136	0,4762
**276**	2	2	10,9	sp|O75569|PRKRA_HUMAN	Interferon-inducible double stranded RNA-dependent protein kinase activator A	1	1,6293	0,4217	0,6546	0,4852
**277**	2	2	9,3	sp|O14979|HNRDL_HUMAN	Heterogeneous nuclear ribonucleoprotein D-like	1	1,0666	0,8783	1,6904	0,4016
**278**	2	2	10,3	sp|O00148|DDX39_HUMAN	ATP-dependent RNA helicase DDX39	2	0,8954	0,8187	0,6918	0,5481
**279**	2	2	15,7	sp|Q9UNL2|SSRG_HUMAN	Translocon-associated protein subunit gamma	2	0,9908	0,6405	1,1695	0,602

**280**	2	2	17,7	sp|Q9UBY9|HSPB7_HUMAN	Heat shock protein beta-7	1	1,2246	0,679	1,0765	0,8743
**281**	2	2	14,3	sp|Q9H444|CHM4B_HUMAN	Charged multivesicular body protein 4b	1				
**282**	2	2	11,4	sp|Q99961|SH3G1_HUMAN	Endophilin-A2	1	1,2706	0,5798	1,2359	0,6932
**283**	2	2	16,5	sp|Q99714|HCD2_HUMAN	3-hydroxyacyl-CoA dehydrogenase type-2	1	1,2589	0,6606	1,3804	0,5337
**284**	2	2	14,5	sp|Q96JY6|PDLI2_HUMAN	PDZ and LIM domain protein 2	1	0,9727	0,6702	1,3677	0,5679
**285**	2	2	8,7	sp|Q8NFI4|F10A5_HUMAN	Putative protein FAM10A5	1	1,4322	0,5203	1,7865	0,3746
**286**	2	2	21,5	sp|P62820|RAB1A_HUMAN	Ras-related protein Rab-1A	4	0,9908	0,4012	0,9908	0,3779
**287**	2	2	20,3	sp|P51149|RAB7A_HUMAN	Ras-related protein Rab-7a	2	0,9908	0,9506	1,0568	0,8574
**288**	2	2	26	sp|P42766|RL35_HUMAN	60S ribosomal protein L35	1	1,2589	0,4122	1,0375	0,7746
**289**	2	2	9,7	sp|P38159|HNRPG_HUMAN	Heterogeneous nuclear ribonucleoprotein G	1	1,2246	0,6813	1,406	0,5323
**290**	2	2	22,5	sp|P30086|PEBP1_HUMAN	Phosphatidylethanolamine-binding protein 1	1	1,3552	0,5726	1,0864	0,856
**291**	2	2	21,9	sp|P30085|KCY_HUMAN	UMP-CMP kinase	1	0,6792	0,5135	0,8241	0,7241
**292**	2	2	14,4	sp|P23396|RS3_HUMAN	40S ribosomal protein S3	1	1,4191	0,5301	0,8318	0,7263
**293**	2	2	14,4	sp|P09497|CLCB_HUMAN	Clathrin light chain B	1	0,8318	0,7384	1	0,9911
**294**	2	2	7,5	sp|P07099|HYEP_HUMAN	Epoxide hydrolase 1	1	1,8197	0,3634	0,7112	0,5544
**295**	2	2	9,2	sp|Q9UL46|PSME2_HUMAN	Proteasome activator complex subunit 2	1				
**296**	2	2	9,5	sp|Q9BVC6|TM109_HUMAN	Transmembrane protein 109	1	1,028	0,9309	1,4723	0,4931
**297**	2	2	27	sp|Q15836|VAMP3_HUMAN	Vesicle-associated membrane protein 3	1	1,3305	0,5856	0,9817	0,9877
**298**	2	2	12,5	sp|Q04760|LGUL_HUMAN	Lactoylglutathione lyase	1	0,6081	0,4312	0,8241	0,719
**299**	2	2	28,9	sp|P63220|RS21_HUMAN	40S ribosomal protein S21	3				
**300**	2	2	24,4	sp|P62854|RS26_HUMAN	40S ribosomal protein S26	1	0,7727	0,6143	1,1482	0,789
**301**	2	2	13,9	sp|P52943|CRIP2_HUMAN	Cysteine-rich protein 2	1	0,6427	0,4655	1,0765	0,8735
**302**	2	2	8,5	sp|P35237|SPB6_HUMAN	Serpin B6	1	0,8872	0,821	0,8017	0,6884
**303**	2	2	11,4	sp|P30084|ECHM_HUMAN	Enoyl-CoA hydratase, mitochondrial	2	0,9908	0,9109	0,8395	0,8503
**304**	2	2	17	sp|P30046|DOPD_HUMAN	D-dopachrome decarboxylase	1	0,5861	0,4058	1,0375	0,9216
**305**	2	2	10,6	sp|P15559|NQO1_HUMAN	NAD(P)H dehydrogenase [quinone] 1	1	1,0186	0,961	1,2589	0,6489
**306**	2	2	34,9	sp|P14854|CX6B1_HUMAN	Cytochrome c oxidase subunit 6B1	1	1,2474	0,6566	1,1912	0,7158
**307**	2	2	6,7	sp|P12236|ADT3_HUMAN	ADP/ATP translocase 3	1	1,1482	0,7673	1,0568	0,8982
**308**	2	2	40,8	sp|Q15847|APM2_HUMAN	Adipose most abundant gene transcript 2 protein	2	1,0186	0,9939	0,929	0,9486
**309**	2	2	14,4	sp|P58546|MTPN_HUMAN	Myotrophin	1	1,4588	0,5027	1,3183	0,5988
**310**	2	2	11,1	sp|P49773|HINT1_HUMAN	Histidine triad nucleotide-binding protein 1	1	0,863	0,6687	1,1912	0,8564
**311**	2	2	7,5	sp|P22061|PIMT_HUMAN	Protein-L-isoaspartate(D-aspartate) O-methyltransferase	1	0,8551	0,7761	1,0375	0,9245
**312**	2	2	7,8	sp|P21291|CSRP1_HUMAN	Cysteine and glycine-rich protein 1	1	1,8535	0,3575	1,2942	0,5958
**313**	2	2	14	sp|P05386|RLA1_HUMAN	60S acidic ribosomal protein P1	2	0,6792	0,5029	0,9204	0,6757
**314**	2	2	7,8	sp|O60888|CUTA_HUMAN	Protein CutA	1	0,6982	0,5919	1,0093	0,9205
**315**	1,82	2	11,5	sp|P00403|COX2_HUMAN	Cytochrome c oxidase subunit 2	1	1,4723	0,4993	1,2246	0,6792
**316**	1,59	1,59	11,6	sp|Q9BS26|ERP44_HUMAN	Endoplasmic reticulum resident protein 44	1	0,955	0,9396	0,8166	0,7047
**317**	1,54	1,54	5,7	sp|P08195|4F2_HUMAN	4F2 cell-surface antigen heavy chain	1	1,2246	0,6888	0,8241	0,722
**318**	1,46	1,46	29,1	sp|P24844|MYL9_HUMAN	Myosin regulatory light polypeptide 9	1	1,4997	0,4826	1,5704	0,4466
**319**	1,37	1,37	15,9	sp|P62318|SMD3_HUMAN	Small nuclear ribonucleoprotein Sm D3	2	0,7798	0,6223	0,9376	0,9791
**320**	1,35	1,35	17,9	sp|P27695|APEX1_HUMAN	DNA-(apurinic or apyrimidinic site) lyase	1				
**321**	1,33	1,33	12,6	sp|Q9NPQ8|RIC8A_HUMAN	Synembryn-A	1	61,9441	0,0173	0,9908	1

Samples were labeled as: siCTRL 1 (CTRL1), 114; siHIF-1α 1 (HIF1), 115; siCTRL 2 (CTRL2), 116; siHIF-1α 2 (HIF2), 117.
Table columns show unused and total ProtScore, percentage of protein sequence coverage (% Cov), accession number and protein name, number of peptides identified above 95% confidence, and ratio and p-value in all siHIF-siCTRL comparisons.

Table S3. Primer sequences used for quantitative real-time PCR analysis

Gene Name	Forward Primer Sequence	Reverse Primer Sequence
HIF-1α	5'-CCATTAGAAAGCAGTTCCGC-3'	5'-TGGGTAGGAGATGGAGATGC-3'
PDK1	5’-CTATGAAAATGCTAGGCGTCTGT-3’	5’-TGGGATGGTACATAAACCACTTG-3’
GAPDH	5’-TGTTGCCATCAATGACCCCTT-3’	5’-CTCCACGACGTAGTCAGCG-3’
APLN	5'-GTCTCCTCCATAGATTGGTCTGC-3'	5'-GGAATCATCCAAACTACAGCCAG-3'
INHBB	5'-GCCTGTGGCTTTACCTGAAACT-3'	5'-CCTGCTCCTGGAAGTACACTTTG-3'
SDHC	5’-CTGTTGCTGAGACACGTTGGT-3’	5’-ACAGAGGACGGTTTGAACCTA-3’
SUCLG2	5'-CAAAAGACCCTAATGTTGTGGGA-3'	5'-TTCAGCAACCATCACCTTGTT-3'
β-actin	5'-GCGCGGCTACAGCTTCAC-3'	5'-GGCCATCTCTTGCTCGAAGT-3'

HIF-1α, hypoxia inducible factor 1, alpha subunit; PDK1, pyruvate dehydrogenase kinase, isozyme 1; GAPDH, glyceraldehyde-3-phosphate dehydrogenase; APLN, apelin; INHBB, inhibin beta; SDHC, succinate dehydrogenase complex, subunit C; SUCLG2, succinate-CoA ligase, GDP-forming, beta subunit.

Supplementary Figure S1. **Induction of GAPDH by $CoCl_2$.** Time-course of GAPDH induction after treatment with 300μM $CoCl_2$. **(a)** Expression of GAPDH mRNA in SF by qRT-PCR. β-actin was used as an endogenous reference. Data are mean±SD of triplicates from 2 independent experiments. **(b)** Western blot image of GAPDH and β-actin, and densitometric analysis of GAPDH protein expression normalized to the β-actin level.

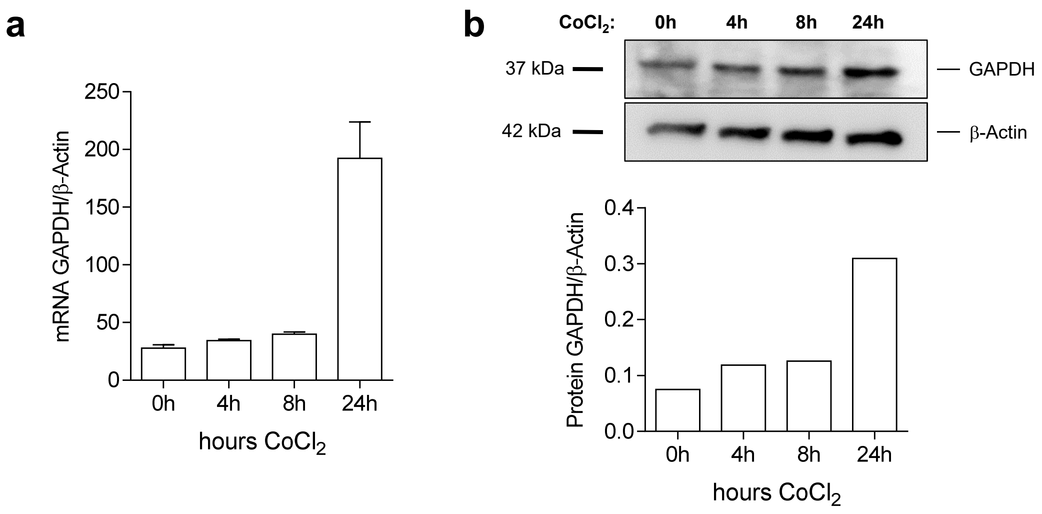

Supplementary Figure S2. Full-length blots used to generate Fig S1.

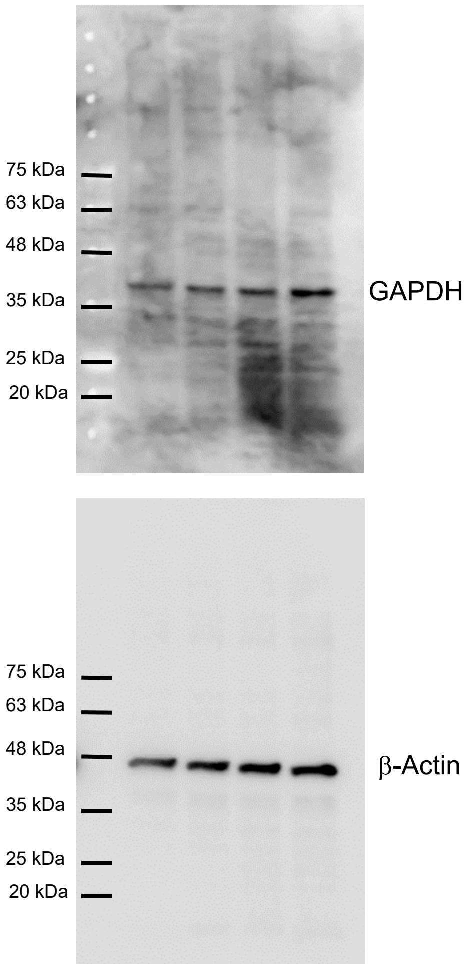

Supplementary Figure S3. Full-length blots used to generate Fig 3.

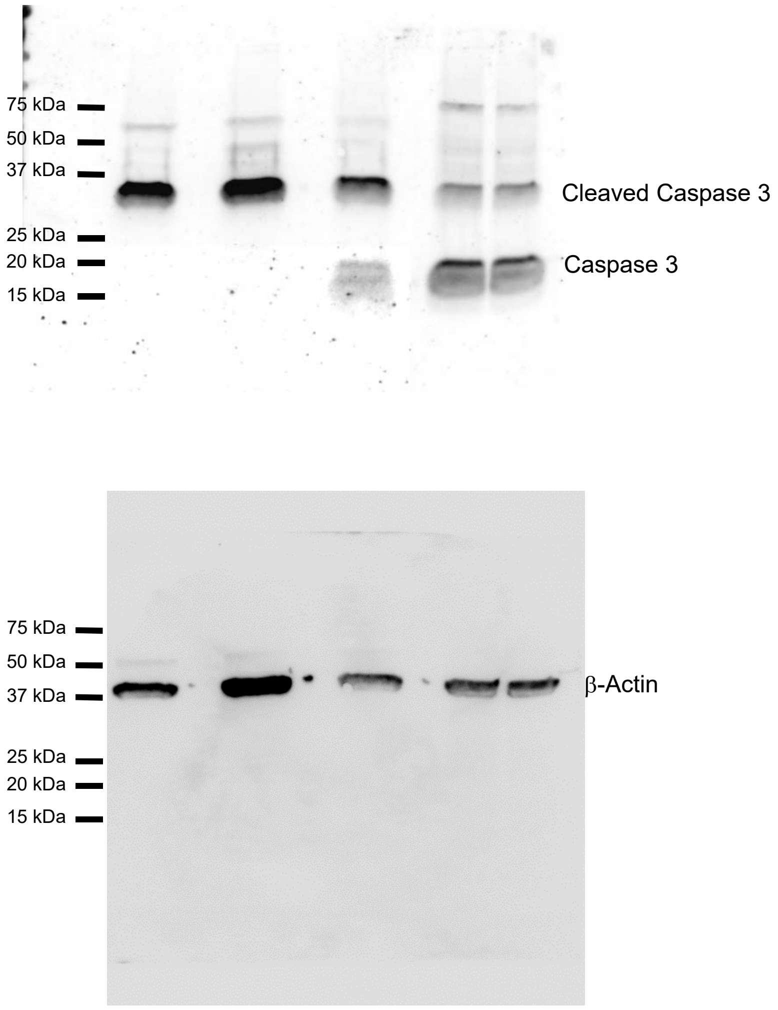

Supplement: Supplementary file 1 — Supplementary information [file 41598_2017_3921_MOESM1_ESM.pdf]
